# Ectopic expression of *Triticum aestivum SERK* genes (TaSERKs) control plant growth and development in *Arabidopsis*

**DOI:** 10.1038/s41598-017-10038-1

**Published:** 2017-09-28

**Authors:** Akanksha Singh, Paramjit Khurana

**Affiliations:** Department of Plant Molecular Biology, University of Delhi, New Delhi, 110021 India

## Abstract

Somatic embryogenesis receptor kinases (SERKs) belong to a small gene family of receptor-like kinases involved in signal transduction. A total of 54 genes were shortlisted from the wheat genome survey sequence of which 5 were classified as *SERK*s and 49 were identified as *SERK-like* (*SERLs*). Tissue- specific expression of *TaSERK*s at major developmental stages of wheat corroborates their indispensable role during somatic and zygotic embryogenesis. *TaSERK* transcripts show inherent differences in their hormonal sensitivities, i.e. *TaSERK2* and *TaSERK3* elicits auxin- specific responses while *TaSERK1*, *4* and 5 were more specific towards BR-mediated regulation. The ectopic expression of *TaSERK1*, *2*, *3*, *4* and 5 in *Arabidopsis* led to enhanced plant height, larger silique size and increased seed yield. Zygotic embryogenesis specific genes showed a differential pattern in *TaSERK Arabidopsis* transgenics specifically in the silique tissues. Elongated hypocotyls and enhanced root growth were observed in the overexpression transgenic lines of all five *TaSERK*s. The inhibitory action of auxin and brassinosteroid in all the *TaSERK* transgenic lines indicates their role in regulating root development. The results obtained imply redundant functions of *TaSERK*s in maintaining plant growth and development.

## Introduction

Somatic embryogenesis (SE) is the developmental reprogramming of somatic cells towards the embryogenic pathway which forms the basis of cellular totipotency in higher plants^1,2^. This unique developmental pathway involves a plethora of characteristic events viz., cellular dedifferentiation, cell division activation, reorganization of physiology and regulation of gene expression patterns^3^. Several genes involved in embryogenic competence have been studied in *Arabidopsis* such as SERK^4,5^, LTP^2^, BBM^6,7^, LEC^8,9^, PKL^10,11^, CLV^12^, WUS^13^, AGL–15^14^ and LEC1–LIKE^15^. Interestingly, a *SERK* related gene, functioning in ancestral conjugate algae, may have been recruited with a novel function similar to SE during evolution from unicellular algae to multicellular plant organisms^16^. In wheat, earlier reports manifested 2,4–D induced SE in the leaf base region^17^ which was further demonstrated to be mediated by Ca^2+^–CaM pathway^18,19^, providing necessary insight into the process of plant embryogenesis.

SOMATIC EMBRYOGENESIS RECEPTOR LIKE KINASE (SERK) first isolated from carrot (*Daucus carota*) embryogenic cells, is considered a characteristic molecular marker for SE in carrot, *Dactylis glomerata* and *Arabidopsis* ^4,5,20^. Since it is expressed in somatic cultures exhibiting close homology with animal and plant receptor kinases, it was named as *somatic embryogenesis receptor kinase* (*SERK*) gene. *SERK* genes belong to a small receptor like kinase family (RLKs) identified in many plant species with five members in *Arabidopsis* ^5^, three in *Zea mays* ^21^, five in *Medicago truncatula* ^22^, four in *Helianthus annus* ^23^, two in *Oryza sativa* ^24,25^, three in *Vitis vinifera* ^26^, three in *Phoenix dactylifera* ^27^ and at least three in *Triticum aestivum* ^28^. In addition, *SERK*-like genes have also been reported in *Poa pratensis* and rice, with eight^29^ and nine members^30^ respectively. *SERK* gene expression in *D*. *carota* appears in embryogenic competent cell cultures and continues to the globular stage of embryos while no expression is detected in non-embryogenic cultures^4^. In *D*. *glomerata*, expression of SERK was reported in leaf segments and continues in shoot apical meristems^20^. Ectopic expression of *AtSERK1* results in enhanced embryogenic cell formation^5^. *AtSERK1* and *AtSERK2* function redundantly in maintaining the development of the male gametophyte^31^. In *P*. *pratensis*, *PpSERK1* expression was high during premeiosis and decreased during meiosis and post-meiotic stages, whereas expression in *PpSERK2* was high from premeiosis to anthesis^29^. Contrastingly in *Z*. *mays*, *SERK* expression was reported in both embryogenic and non-embryogenic callus cultures^21^. In *O. sativa, OsSERK1* is expressed in phytohormone sensitive tissues where it mediates defense signal transduction while *OsSERK2* is expressed in all other plant organs^24,25^. Recently in *P*. *notatum*, *PnSERK2* was correlated with the onset of apomixis as it showed expression in nucellar cells at the meiosis stage of the apomictic genotype^32^. These studies substantiate that the *SERK* genes play a crucial role during embryogenesis and have functional relevance in other facets of plant growth and development.

The present study was undertaken to gain insight into the expression and functional significance of *SERK* genes in wheat, *T*. *aestivum*. To achieve this, we cloned and characterised five *TaSERK*s and raised the overexpression (OE) transgenics in *Arabidopsis*. Here, we report their sequence analysis, structural organisation, phylogenetic relationship and expression analysis in different zygotic and somatic tissues of wheat. We also demonstrate the effect of auxin and brassinosteroid on root growth in *TaSERK* OE transgenic lines. Differential expression analysis of other embryogenesis related genes in OE transgenics demonstrates the possible role of *TaSERKs* in embryogenesis and seed development. Constitutive expression of *TaSERKs* in *Arabidopsis* results in enhanced hypocotyl length, plant height, altered silique size and seed yield.

## Experimental Procedures

### Plant material and growth conditions

#### *Triticum aestivum*

Seeds of *T*. *aestivum* var. PBW343 were surface sterilised with 4% sodium hypochlorite for 30 min and inoculated on water soaked cotton bed and covered with Klin wrap for maintaining humidity. The seeds were grown under culture room conditions at 28 °C, with a daily photoperiodic regime of 16 h light and 8 h dark cycle where light was provided by fluorescent tubes (Philip TL 40 W/ 54) at a fluence rate of 80–100 µmol m^−2^s^−1^, as per experimental requirements. 13–d–old wheat seedling tissues were used for detailed experiments according to the protocol described earlier^17,19^. The zygotic tissue of wheat was raised, collected from field–grown plant, and immediately frozen in liquid nitrogen and stored at −80 °C until use. Embryogenic and non–embryogenic calli were raised as described previously^33^ and for auxin (2,4–D) and brassinosteroid (epi–BL) leaf base induction treatment, experimental method was carried out as described earlier^34^.

#### *Arabidopsis thaliana*

To raise OE transgenic lines in *Arabidopsis thaliana* ecotype Col–0, plants were grown in pots containing Soilrite (Kelpirite, Bangalore; 1:1:1 ratio of Vermiculite, Perlite and *Sphagnum* moss) supplemented with OS medium^35^ in a culture room under 80–100 µmol m^−2^ s^−1^ at 22 ± 1 °C with 16 h /8 h light and dark photoperiod regime. *TaSERKs*: pMDC32 was transformed in *Agrobacterium* and transgenic plants were generated as described previously^36^.

### Genome wide analysis of SERKs in wheat

To identify homologues of SERK in wheat (*T*. *aestivum*), the National Centre for Biotechnology Information (NCBI, https://blast.ncbi.nlm.nih.gov/Blast.cgi), the *Arabidopsis* information resource (TAIR, http://www.arabidopsis.org/Blast/index.jsp) and Rice Genome Annotation Project (RGAP, http://rice.plantbiology.msu.edu/analyses_search_blast.shtml) databases were used. The deduced amino acid sequences of the known SERK proteins was employed to search for other homologues in wheat by using the TBLASTN program. The redundant sequences were removed using CLC main workbench software. The search was based on the presence of the characteristic features of SERKs, i.e. presence of SPP motif and the C–terminal domain. Additionally, we also made an attempt to identify the SERK homologues from wheat genome survey sequences. For this, the wheat genome sequences were downloaded from URGI sequence repository (http://wheat-urgi.versailles.inra.fr/) which was then Blast searched (blastn version 2.2.6) using CDS sequences of the already known wheat SERKs as a query. Sequences obtained from the BLAST were then utilised for protein prediction using GENSCAN version 1.0^37^. Protein sequences were retrieved and analysed using TMHMM Server v. 2.0 (http://www.cbs.dtu.dk/services/TMHMM/) for the identification of transmembrane helices also. The sequences were then aligned for the search of SPP motif to identify SERKs in wheat.

### *In silico* analysis of TaSERKs

The nucleotide and protein sequences of cloned TaSERKs were analysed using Gene Runner Program 3.04 (http://www.genenames.com). Deduced protein sequences were used to decipher domain organization using SMART (http://smart.embl/heidelberg.de/). The nucleotide and amino acid sequence were searched to obtain homologues from other plants as well (using NCBI database BLAST program). Phylogenetic tree of TaSERKs was generated using the neighbor-joining (NJ) method in MEGA (version 6) software program.

### RNA Isolation and cDNA synthesis

RNA from wheat embryogenic calli and overexpression *Arabidopsis* transgenics were isolated by RNeasy Plant mini kit (Qiagen, Germany) according to the manufacturer’s instructions followed by DNase–I treatment for removal of genomic DNA contamination. For cDNA synthesis, 2 µg RNA was used for the amplification and the PCR conditions was followed according to the manufacturer’s instructions using Superscript III one–step RT–PCR (Invitrogen, USA). The cDNA synthesised was used as a template for further amplification of *TaSERKs*. For real–time expression analysis, cDNA was prepared from 2 µg RNA using High capacity cDNA archive kit (Applied Biosystems, USA)^38^. Primers used for real–time PCR analysis are listed in Supplementary Table S2.

### Isolation of full–length cDNA of *TaSERKs* (*TaSERK1*, *2*, *3*, *4*, 5)

For the amplification of full length cDNA of *TaSERKs*, RNA was isolated from the wheat embryogenic calli and cDNA prepared by one step RT–PCR using Superscript® III First–Strand Synthesis System RT–PCR kit (Invitrogen, USA) was used as a template for the amplification of *TaSERK* genes using Phusion High Fidelity Taq polymerase (Finnzymes). The thermal cycling condition was as follows: initial denaturation at 98 °C for 30 s followed by amplification for 35 cycles at 98 °C for 10 s, annealing at 62 °C for 30 s, extension at 72 °C for 1 min and a final extension at 72 °C for 7 min. Each of the full length amplified products of all *TaSERKs* obtained were then cloned individually in pDRIVE vector (PCR cloning kit, Qiagen, Germany) and entry vector pENTR^TM^/D–TOPO (Invitrogen Inc. USA) as described previously^39^. Primers used for above cloning were listed in Supplementary Table S2.

### Hypocotyl assay

For hypocotyl assay, seeds were germinated on half-strength MS medium supplemented with 2% sucrose and 0.8% agar in Petri plates kept in growth room at 22 ± 1 °C. After 7 d of growth, the hypocotyl length of ten seedlings each from the WT and *TaSERKs* overexpression (*TaSERKs*-OE) lines were measured.

### Root growth assay

For examining the effect of brassinosteroid (epi–BL) and auxin (2,4–D) on root growth assay, seedlings were grown on half-strength MS medium supplemented with 2% sucrose and 0.8% agar in plates kept vertically for 3 d at 22 ± 1 °C. Seedlings were then transferred on to a fresh MS medium supplemented with 24–epibrassinolide and 2,4–D (Sigma, St Louis, MO, USA) at different working concentrations and placed vertically under normal culture conditions for 4 d. The root length was measured on the fifth day of transfer and was compared to control seedlings. All experiments were done in triplicates, and the values presented in the data are mean of these experiments, and standard error was calculated.

### Statistical analysis

Data of 10–15 seedlings for root growth measurement, 20–25 seedlings for hypocotyl elongation assay, 10–20 plants for morphological and phenotypic evaluation from WT and transgenic plants were collected. Student’s t-test was calculated for significant differences between WT and transgenic lines. A p-value of 0.05 was considered significant.

## Results

### Sequence analysis of *TaSERK* genes and relationship with other family members

Our previous study (Singla *et al*.)^28^ had identified three *TaSERKs* from *T*. *aestivum*, one of which was specifically isolated from an auxin induced cDNA library^40^ (*TaSERK3*). In the present study, we identify additional SERK genes in the wheat genome from sequence analysis through BlastN, Blastp, TBlastX search of ESTs and cDNA clones using NCBI, RGAP, KOME databases and analysed the sequences after multiple sequence alignment by the CLC main workbench program. From the above sequence analysis, we identified five SERKs which were named as TaSERK1 (Accession no. AK333001), TaSERK2 (Accession no. AK3336771), TaSERK3 (Accession no. BT009223), TaSERK4 (Accession no. Ta76279_4565) and TaSERK5 (Accession no. BT009426). The sequences were then confirmed by amplifying full length cDNAs and verified by sequencing (Supplementary Fig. S1).

*TaSERK1* harbours a 168 bp 5′UTR and 306 bp 3′UTR; *TaSERK2* has an 110 bp and 307 bp long 5′ and 3′UTR; *TaSERK3* has a 133 bp and 244 bp 5′ and 3′UTR; *TaSERK4* has a 397 bp and 272 bp 5′ and 3′UTR and *TaSERK*5 contains a 130 bp 5′UTR and 284 bp 3′UTR, respectively. TaSERK1, TaSERK2, TaSERK3, TaSERK4 and TaSERK5 encode proteins of 628, 623, 624, 474 and 628 amino acids with predicted molecular weight of 68.94 kDa, 68.48 kDa, 68.76 kDa, 52 kDa and 69.07 kDa, respectively. Sequence analysis revealed that among the TaSERK members, TaSERK1 shows the highest identity with TaSERK4 (92%) and TaSERK5 (98%). Amongst *Arabidopsis* AtSERK members, TaSERK1 is closest to AtSERK2 (85%). TaSERK2 shows closest identity with TaSERK3 (91%) and with AtSERK2 (85%). In addition, TaSERK3 was found to be closest to TaSERK4 and TaSERK5, TaSERK4 with TaSERK1 and TaSERK5, and TaSERK5 with TaSERK1 and TaSERK4, respectively.

Multiple sequence alignment of the deduced amino acid sequences of the TaSERKs and OsSERKs gene family from rice (Supplementary Fig. S2) indicated that TaSERKs are similar to OsSERK1 and OsSERK2, sharing characteristic domain features of RLKs, including five leucine–rich repeats (LRR), a SPP (ser–pro–pro) motif (a hallmark feature of SERK gene family), a transmembrane domain and a serine/threonine kinase domain at the carboxyl terminus responsible for phosphorylating downstream proteins^30^. Detailed domain analysis of TaSERK proteins displayed the presence of a leucine zipper region (Supplementary Fig. S3). The leucine zipper sequence is represented from position 37–58 in TaSERK1, from 29–50 in TaSERK2, from 29–50 in TaSERK3 and from 37–58 in TaSERK5. Only TaSERK4 was found to lack this domain. Additionally, a putative protein kinase ATP–binding site is present in the kinase domain at position 311–333 in TaSERK1, 305–327 in TaSERK2, 304–326 in TaSERK3, 156–178 in TaSERK4 and 311–333 in TaSERK5. A Ser/Thr kinase active–site signature in subdomain VI at position 428–440 in TaSERK1, 422–434 in TaSERK2, 424–436 in TaSERK3, 273–285 in TaSERK4 and 428–440 in TaSERK5 is indicative of serine/threonine kinases.

**Figure 1:**
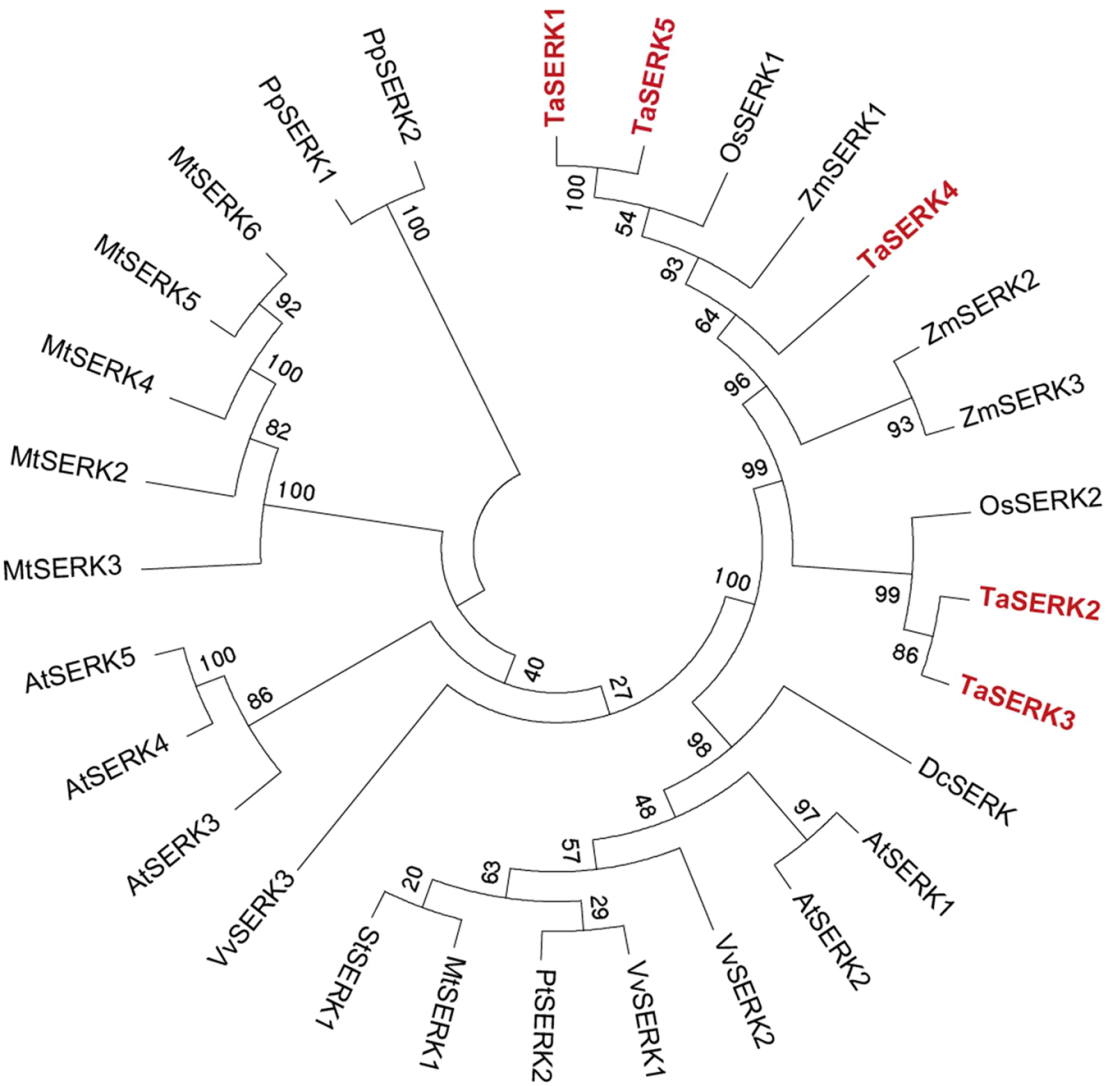
Phylogenetic relationship of TaSERKs. Tree was constructed by neighbour-joining (NJ) method using MEGA (version 6) software with its homologs across various plant species. Bootstrap values out of 100 replicate data sets have been displayed at the branch nodes.

Phylogenetic analysis revealed that TaSERKs clustered together with SERKs in other monocot species, with SERKs in dicot plants clustering separately (Fig. 1). It is evident from the tree that TaSERK1 and TaSERK5 are closest to OsSERK1 (AK103038), TaSERK4 is closest to ZmSERK1 (CAC37640), and TaSERK2 and TaSERK3 are closely related to OsSERK2 (AK099777).

### Differential expression of *TaSERK* genes

**Figure 2:**
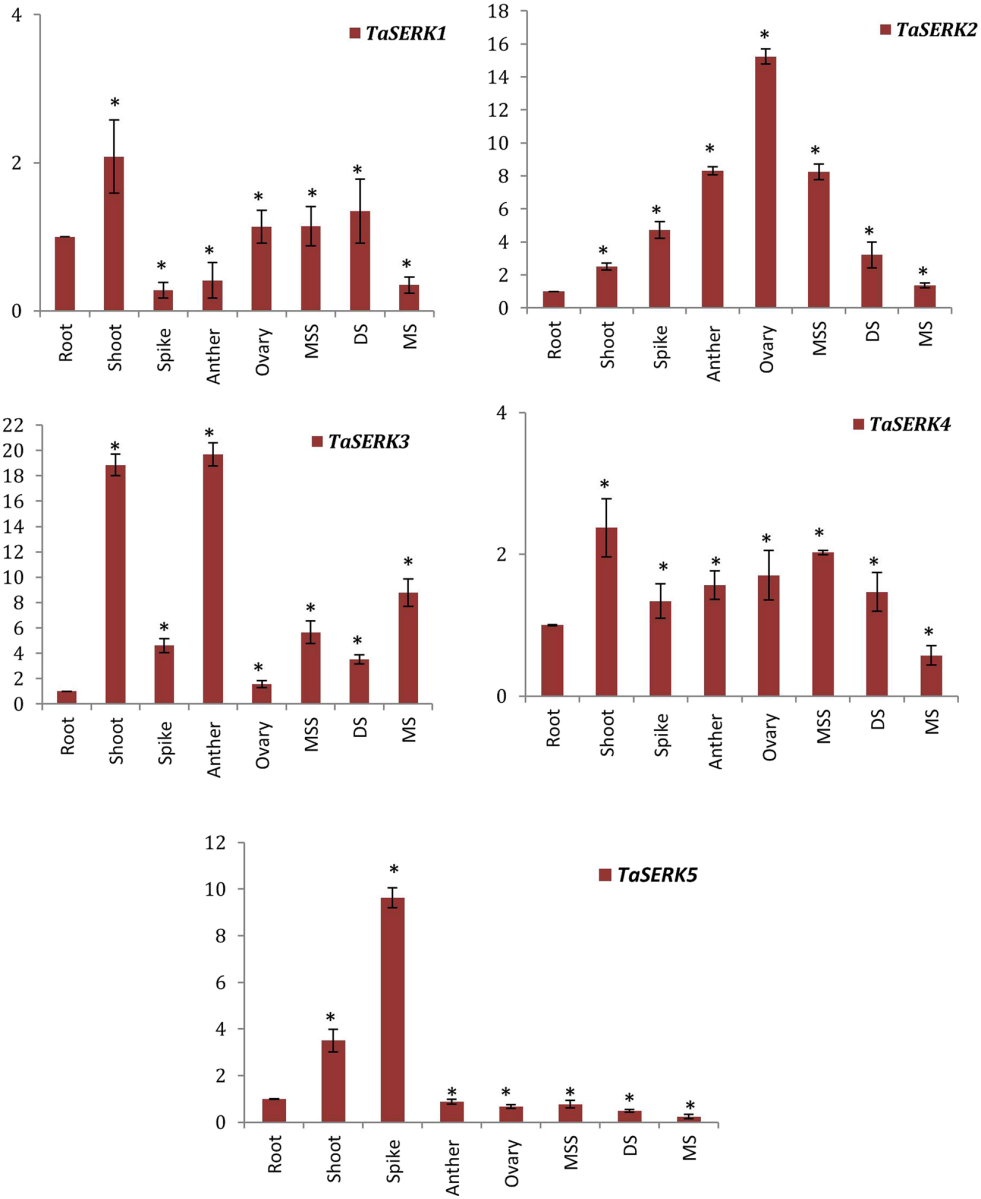
Expression profiles of *TaSERK1*, *2*, *3*, *4* and *5* by qRT-PCR. cDNAs normalized to housekeeping gene, *ACTIN*, in different tissues. The error bars represent mean ± SD of two biological replicates, each analysed with three technical replicates. Asterisks above error bars represent the significance levels (Students *t*-test; *p value ≤ 0.05).

Expression profile of *TaSERK* genes in vegetative tissues (root and shoot) and zygotic tissues viz., spike, anther, ovary, milky stage of seed (MSS), developing seed (DS) and mature seed (MS) revealed a vast range of expression patterns in wheat. The expression of *TaSERK1* and *TaSERK4* up-regulated by 2-fold in shoots compared to the root tissue whereas expression in other zygotic tissues was not significantly increased (Fig. 2). The nearly similar expression profile of *TaSERK1* and *TaSERK4* suggests that they may be functionally overlapping and redundant in action. *TaSERK2* expression was up-regulated in the ovaries by 15-fold followed by an 8-fold change in the anther and MSS as compared to the root tissue suggests its predominant role during zygotic embryogenesis. High expression of *TaSERK3* was observed in the shoot (18-fold) and zygotic tissues such as anthers by 20-fold change, followed by an 8-fold change in MS, then 5-fold in MSS, spike (4-fold), as compared to the roots. The significant differential expression of *TaSERK3* indicates its role during both somatic and zygotic embryogenesis. Expression of *TaSERK*5 up-regulated in a vegetative tissue, shoots by 4-fold as well as in zygotic tissue, spikes by 9-fold whereas a low level of expression in other zygotic tissues indicates that *TaSERK5* might play a rather specific role during zygotic embryogenesis. Therefore, differential expression of *TaSERK* gene indicates a higher complexity of this gene family in the functional aspects of plant development.

**Figure 3:**
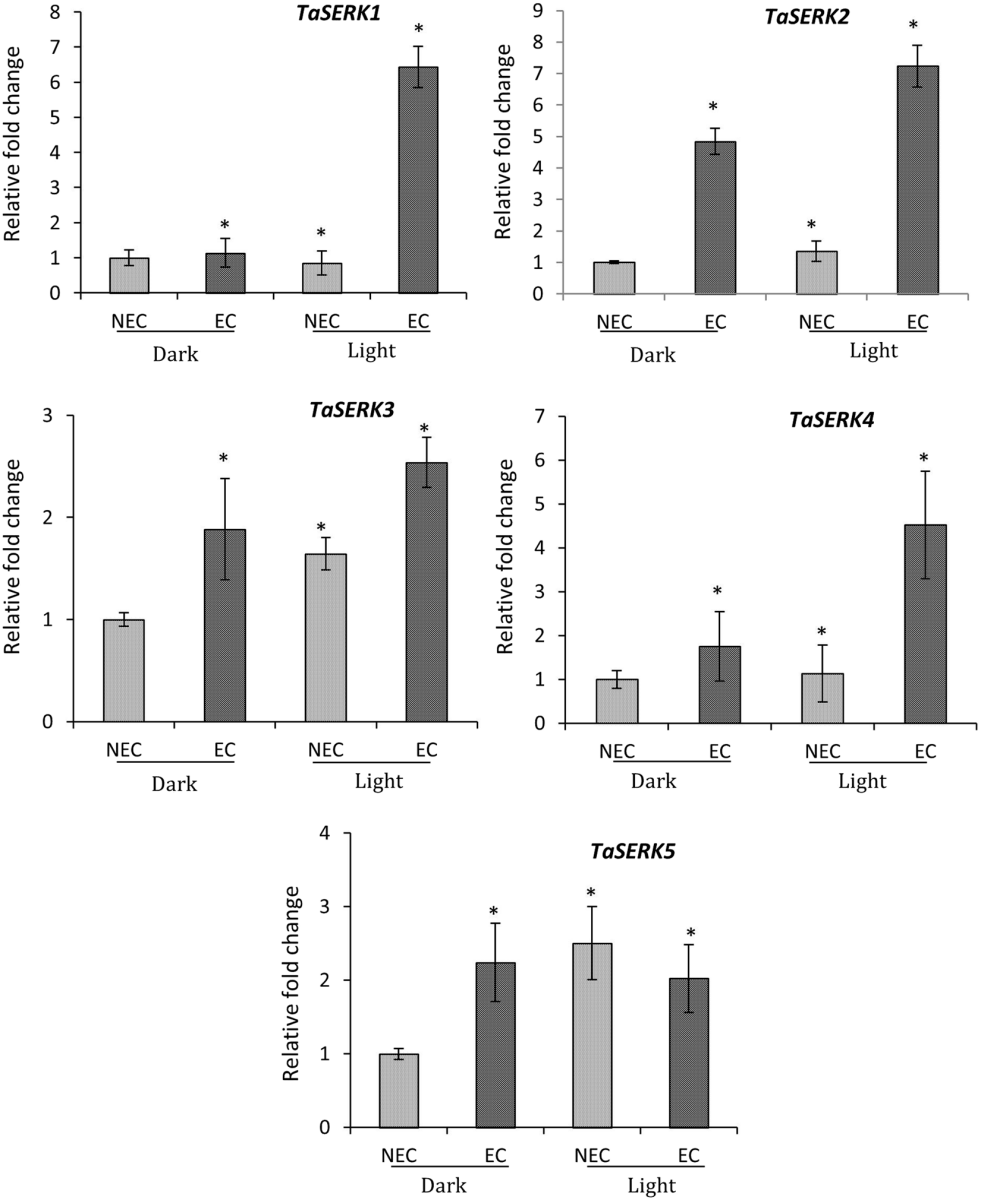
Expression analysis of *TaSERK1*, *2*, *3*, *4* and *5* in wheat embryogenic and non-embryogenic callus. cDNAs normalized to housekeeping gene, *ACTIN*, in different tissues grown under dark and light culture conditions, respectively. The error bars represent mean ± SD of two biological replicates, each analysed with three technical replicates. Asterisks above the bars represent the significance levels (Students *t*-test; *p value ≤ 0.05).

The expression patterns of *TaSERK1*, *2*, *3*, *4* and *5* were examined in wheat embryogenic callus (EC) and non-embryogenic callus (NEC) grown under dark and light culture conditions (Fig. 3). *TaSERK1* (6-fold), *TaSERK*2 (7-fold), followed by *TaSERK*4 (4-fold) and *TaSERK3* (2.5-fold) was found to be up-regulated significantly in EC grown under light conditions relative to NEC. Under light conditions, *TaSERK5* was found to be down-regulated in EC and up-regulated only in NEC (2.5-fold), however, under dark culture conditions except for *TaSERK2* and *TaSERK3* which showed up-regulation in EC, all other *TaSERK1*, *3* and *5* was down-regulated in EC relative to NEC.

### Hormonal sensitivity of TaSERKs

The expression levels of *TaSERK1* and *TaSERK5* (Supplementary Fig. S4) showed BR-mediated up-regulation by 3-fold in treated leaf base explants followed by *TaSERK4* and *TaSERK2* which was up-regulated by 2 fold whereas lower expression was observed in *TaSERK3*. In the presence of 2,4–D only *TaSERK2* was up-regulated ≥2.5 fold as compared to other *TaSERKs*. Therefore, this data suggests that *TaSERK1*, *TaSERK4* and *TaSERK5* are preferentially BR-regulated while *TaSERK2* is preferentially responsive to 2,4-D.

### Generation of overexpressing *TaSERK1*, *2*, *3*, *4* and *5 Arabidopsis* transgenics

To decipher the functional role of *TaSERK* genes *in planta*, each of the five *TaSERK* cDNAs were independently fused in the OE Gateway vector pMDC32 under the control of CaMV 35 S promoter to generate overexpressing transgenic lines of *TaSERK1*, *2*, *3*, *4* and 5 in *Arabidopsis*. All *TaSERK* OE transgenics were confirmed by PCR using *hptII* and (GSP) gene-specific primers (Supplementary Fig. S5) and selected lines were grown to the homozygous stage as described earlier^36^. Transcript levels of selected transgenic lines of *TaSERK1*, *2*, *3*, *4* and 5 were examined by real-time PCR analysis which exhibited variation in the expression level with respect to WT (Supplementary Fig. S6). Three independent lines from each *TaSERK* transgenic were selected for further analysis on the basis of transcript levels and sufficient seed availability.

### Hormone responsive root growth of *TaSERKs* OE transgenics

**Figure 4:**
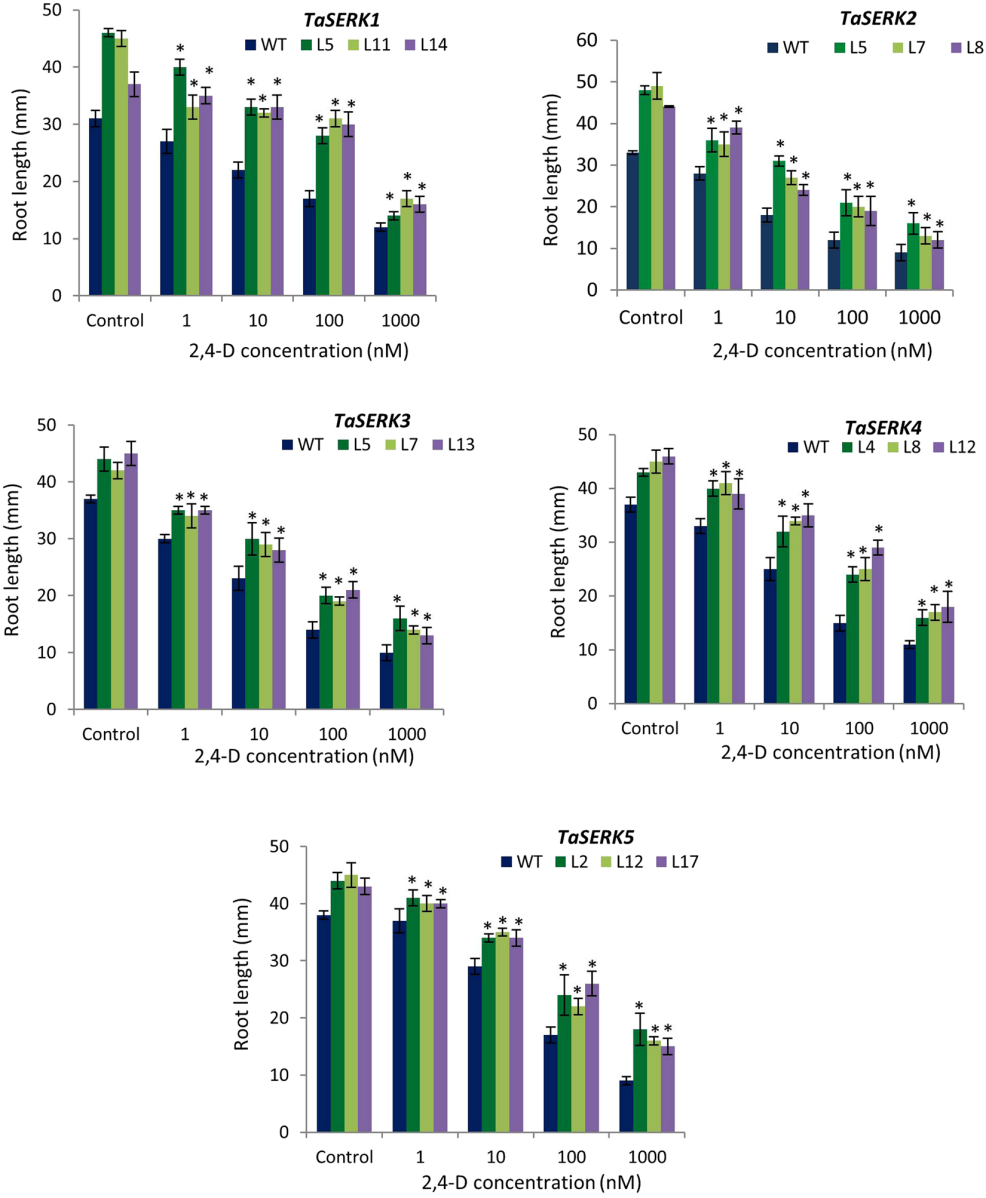
Root growth elongation assay of *TaSERK1*, *2*, *3*, *4* and *5* OE plants under 2,4-D treatment. Histograms represent the root length of WT and *TaSERKs*-OE 7-d old seedlings at different concentrations of auxin supplemented medium. Values are mean ± SE for 10 seedlings each. The asterisks (*p ≤ 0.05) indicate statistically significant differences between WT and transgenic lines.

The root phenotype of OE *TaSERK* transgenic and WT *Arabidopsis* plants in the presence of different plant hormones was examined. The results showed that with increasing concentration of auxin (2,4-D), the root length of *TaSERK1* transgenic lines (L5, L11 and L14) gradually decreased (Supplementary Fig. S7) whereas under control conditions, root growth of *TaSERK1* was significantly longer than WT (Fig. 4A). Similar trends were also observed in other *TaSERKs 2*, *3*, *4* and 5 transgenics in the presence of auxin (Fig. 4B–E).

**Figure 5:**
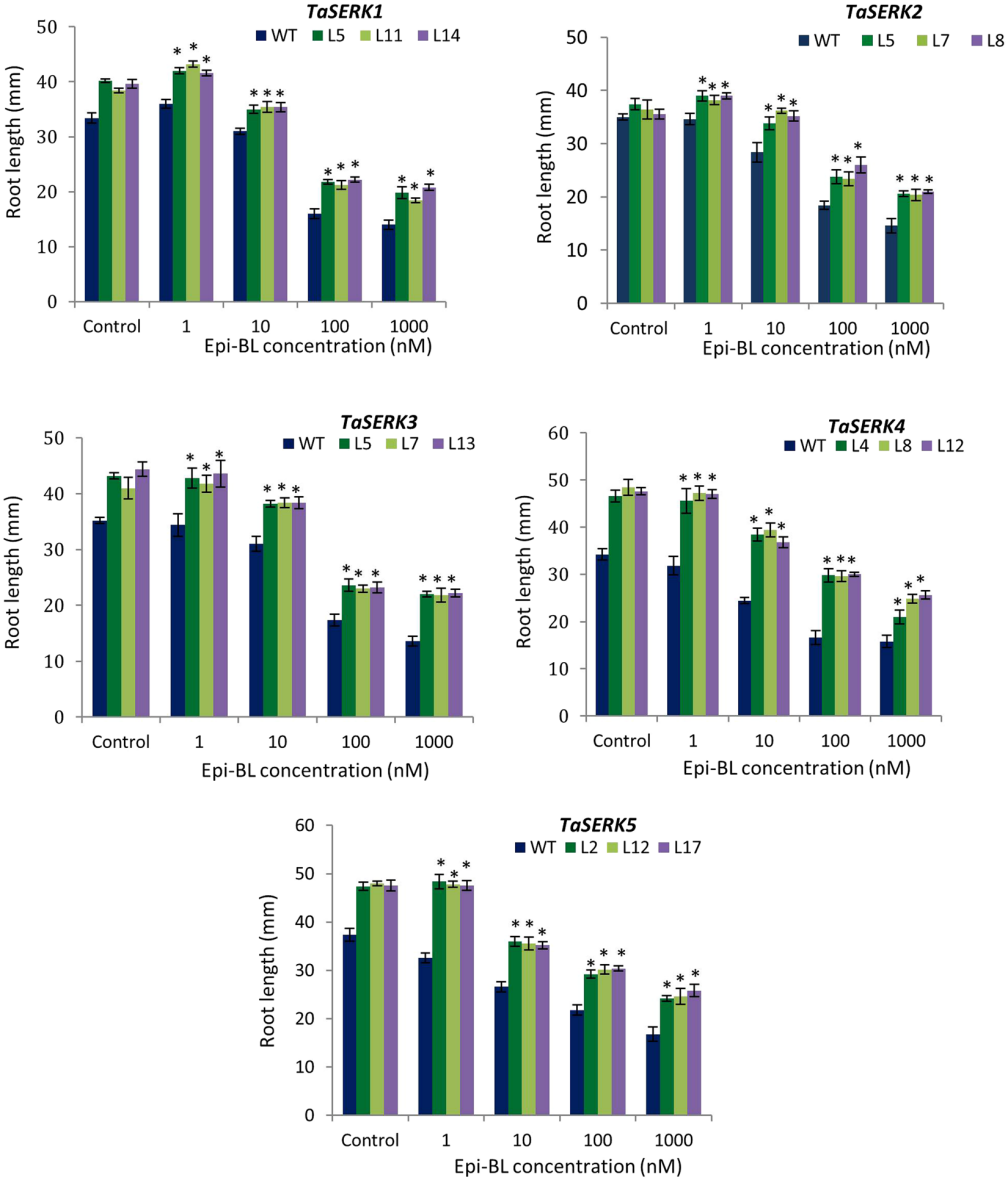
Root growth elongation assay of *TaSERK1*, *2*, *3*, *4* and *5* OE plants under epi-BL treatment. Histograms represent the root length of WT and *TaSERKs*-OE 7-d old seedlings at different concentrations of BR supplemented medium. Values are mean ± SE for 10 seedlings each. The asterisks (*p ≤ 0.05) indicate statistically significant differences between WT and transgenic lines.

We also examined the effect of brassinosteroid (24 epi-BL) on the root growth of *TaSERK* transgenics compared to WT *Arabidopsis* seedlings (Supplementary Fig. S8). Here the root length of *TaSERK1* transgenic lines (L5, L11 and L14) increased at 1 nM 24 epi-BL whereas higher concentrations reduces the root length (Fig. 5A). Similarly, the root length of the other *TaSERK* transgenics (*TaSERK 2*, *3*, *4* and 5) also exhibited same response to different concentrations of epi-BL (Fig. 5B–E). Thus, the above result shows that *TaSERK*-OE plants are sensitive to the inhibitory effect of auxin and BR in a dose- dependent manner.

### Enhanced hypocotyl elongation upon OE *TaSERKs*

**Figure 6:**
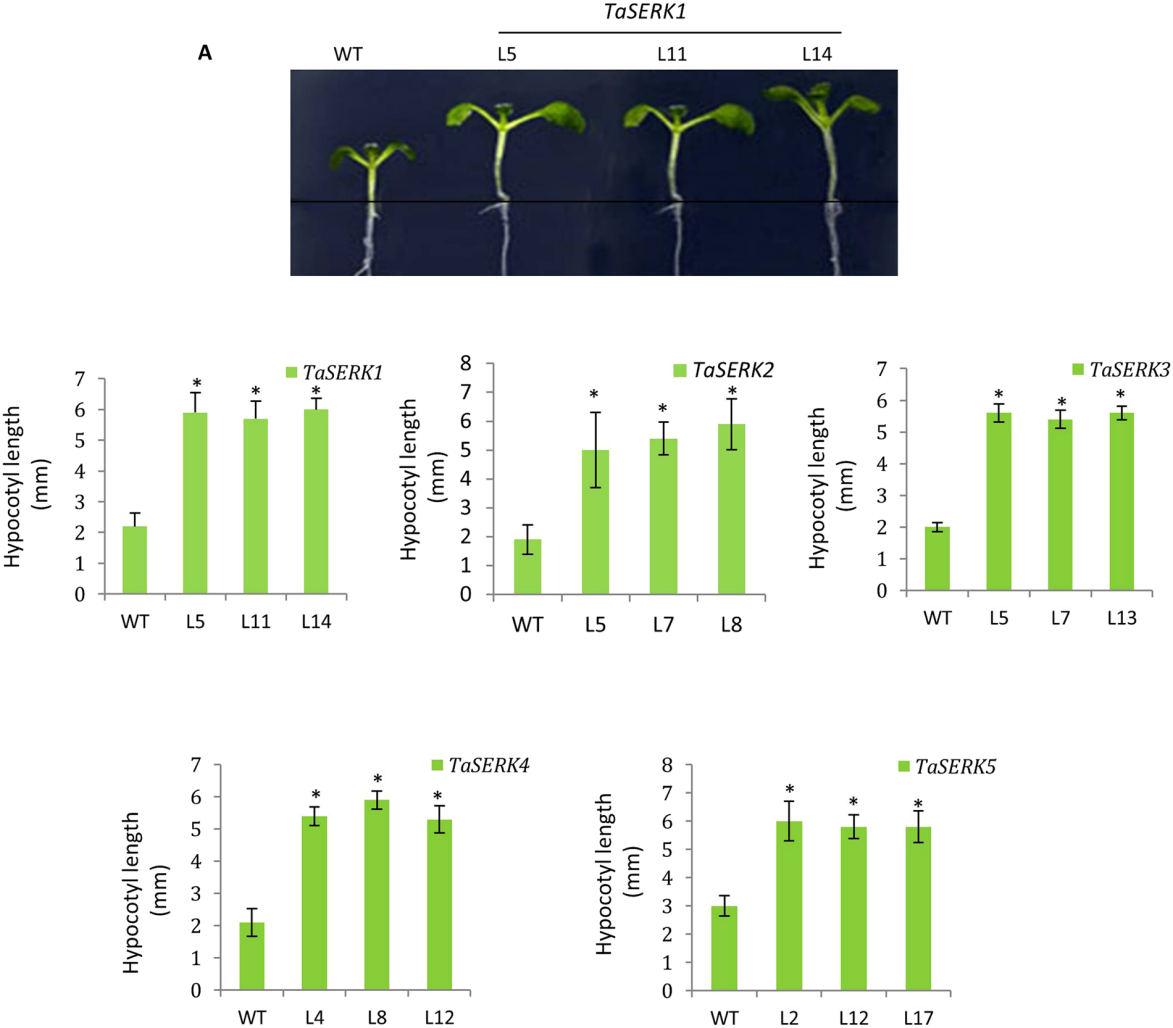
Hypocotyl elongation assay. (**A**) Phenotype of hypocotyl elongation between OE *TaSERK* transgenics and WT *Arabidopsis* seedlings grown on MS medium for seven days. (**B**) Histogram representing the hypocotyl length of WT and *TaSERK* transgenics. Graph plotted taking ± SE of twenty seedlings for each *Arabidopsis* lines. The asterisks (*p ≤ 0.05) indicate statistically significant differences between WT and transgenic lines.

*Arabidopsis TaSERK* transgenic and WT seedlings grown under control culture conditions of 16 h light and 8 h dark photoperiod for seven days showed enhanced hypocotyl length in all the *1*, *2*, *3*, *4* and 5 transgenic lines (Fig. 6A). Measurement and statistical analysis of the hypocotyl length (Fig. 6B) indicated significant differences between all five *TaSERK* transgenic seedlings compared to the WT. The above results clearly demonstrate that OE of *TaSERKs* in *Arabidopsis* promotes hypocotyl elongation under light conditions.

### Constitutive expression of *TaSERK* genes enhance plant growth and seed yield

**Figure 7:**
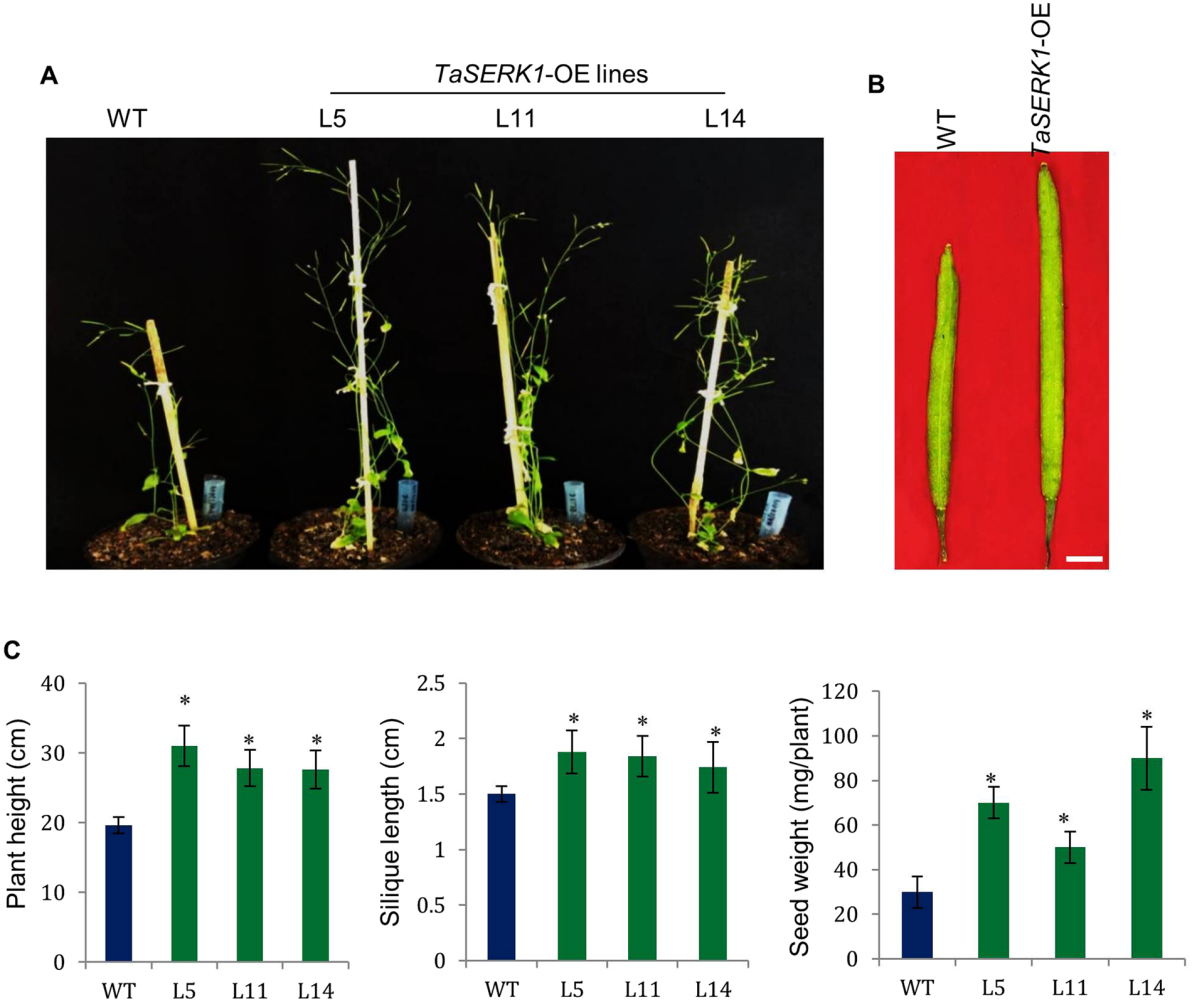
Morphological analysis of *TaSERK1* OE lines in *Arabidopsis thaliana*. (**A**) *TaSERK1* showed increase in plant height as compared to the Col-0 WT grown under 16 h light and 8 h dark culture condition. (**B**) Two month old *TaSERK* lines had larger siliques than WT. (**C**) Graphical representation of silique length, total number of siliques per plant and seed weight per individual plant (N = 10). Data represents mean ± SE. The asterisks (*p ≤ 0.05) indicate statistically significant differences between WT and transgenics.

In the present study, constitutive expression of *TaSERKs* in *Arabidopsis* resulted in an overall increase in plant growth and productivity. The effect of OE of *TaSERKs* on plant growth was monitored during the course of development. The *TaSERK1* OE transgenic lines (L5, L11 and L14) showed increased plant height compared to the wild–type (Fig. 7A) after 30 days of germination. Elongation continues in the *TaSERK1*-OE lines whereas it ceases in WT after 40 days. The ectopic expression of *TaSERK1* in *Arabidopsis* also results in larger silique size as well as an increase in the number of siliques per plant with respect to WT (Fig. 7B). This difference is further reflected in the seed weight which showed a significant increase in seed yield per plant in different transgenic lines (Fig. 7C). However, no significant difference was observed in the size of the seed (Supplementary Table S1) between WT and transgenics, implying that the enlarged silique size was due to a greater number of seeds per plant. The difference were also observed in the leaf morphology and rosette leaf numbers, with rosette leaf numbers being higher in WT compared to the transgenics (Supplementary Table S1). Morphological differences of the other *TaSERK* OE transgenics were also measured (Supplementary Figs S9 and S10 and Supplementary Table S1). Here, we observed that all the *TaSERK* transgenic plants demonstrate an overall increase in plant height, larger siliques, an increased number of siliques per plant and increased seed yield when compared to the WT. No appreciable differences, however, was observed in the length of the siliques in *TaSERK5* transgenics; therefore the increase in seed yield here could be attributed to an increase in the number of siliques per plant examined (Supplementary Table S1). Thus, the above similarity in morphometric analyses for all the *TaSERK* transgenic plants can be attributed to the high sequence similarity and functional redundancy between the five *TaSERKs*.

### Expression of various zygotic genes in *TaSERK* -OE transgenics

**Figure 8:**
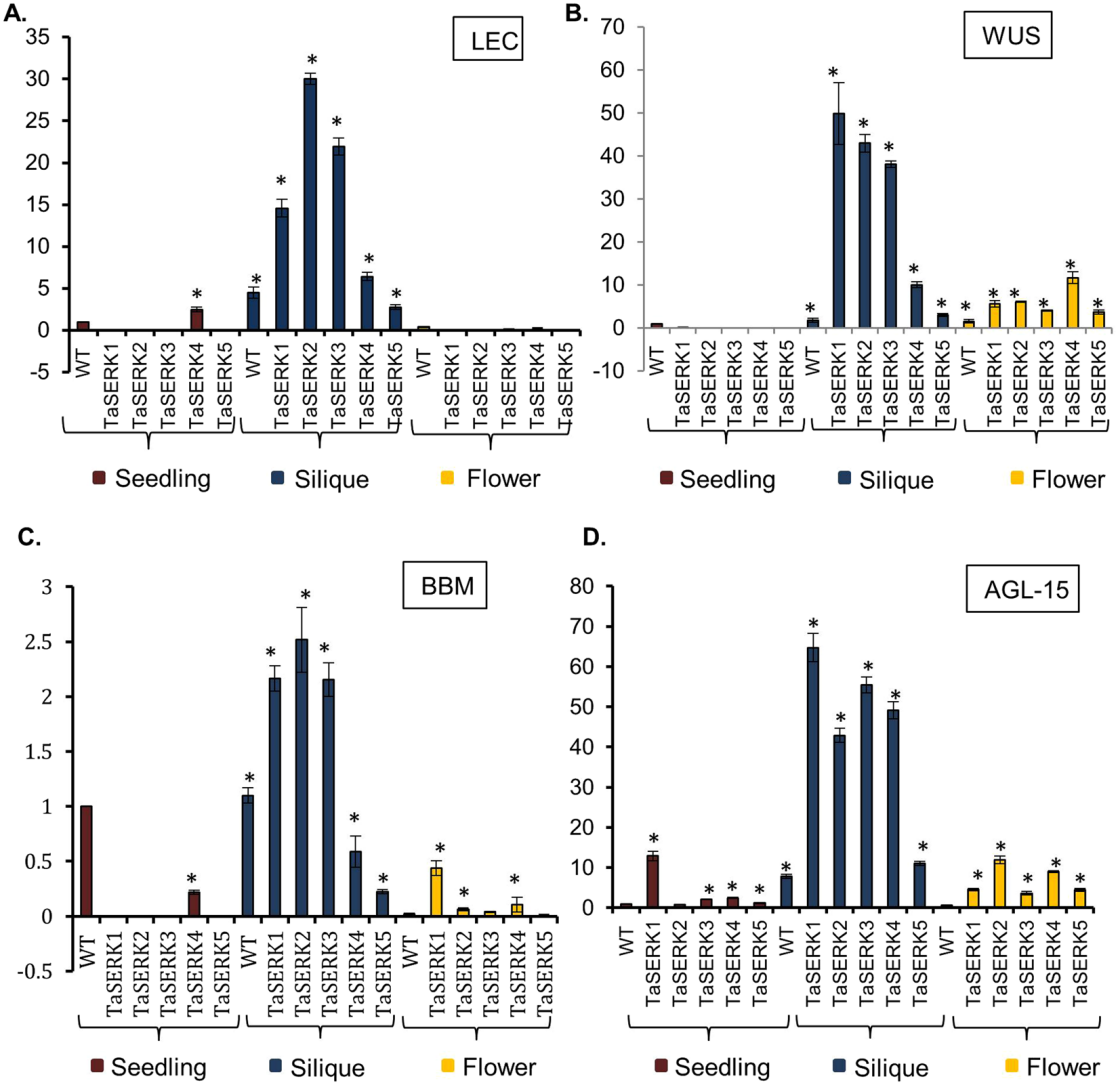
Expression profile of zygotic genes (**A**) *LEC* (**B**) *WUS* (**C**) *BBM* (**D**) *AGL-15* in seedlings, siliques and flower tissue of WT and *TaSERKs*-OE *Arabidopsis* plants. Values were normalised to *Arabidopsis ACTIN* gene expression. The error bars represent mean ± SE of two biological replicates, each analysed with three technical replicates. The asterisks (*p ≤ 0.05) indicate statistically significant differences between WT and transgenics.

To gain insight into the expression profile of known embryogenesis related genes in *TaSERK* OE *Arabidopsis* plants, real-time quantitative PCR was performed to examine the transcript levels of *LEC1*, *WUS*, *BBM* and *AGL–15* zygotic genes. Interestingly, while no expression of *LEC1* gene was observed in either the seedling tissues or in the flower tissues of *TaSERK* -OE plants, the expression of these genes was significantly higher in silique tissues of *TaSERK* transgenics. The *LEC1* gene was markedly up-regulated in siliques, with *TaSERK2-*OE and *TaSERK*3-OE plants exhibiting ≥20–30 fold change, and 15-fold-change in *TaSERK1*–OE plants in comparison to WT (Fig. 8A) however, *TaSERK4*–OE and *TaSERK*5–OE plants showed lower levels of expression as compared to *TaSERK1*, *2* and *3*. A significant change in the expression of the *WUS* gene was observed in the silique tissues compared to seedling and flower tissues of *TaSERK* -OE plants with respect to WT (Fig. 8B). In contrast to the expression of *LEC1*, WUS expression was detected in flower tissues of all *TaSERK* transgenic plants compared to the WT. The expression level analysis of *BBM* (Fig. 8C) and *AGL–15* (Fig. 8D) in different tissues of *TaSERK*-OE transgenic plants, displayed higher expression of both these genes in the silique tissues compared to their meager increase in seedling and flower tissues (Fig. 8C,D). *BBM* expression was highest in *TaSERK2*–OE followed by *TaSERK1* and *TaSERK3*–OE with only small differences observed in *TaSERK*4 and *TaSERK*5–OE plants. *AGL–15* expression levels increased significantly in silique tissues of OE lines of *TaSERK1*, *2*, 3 and *4 Arabidopsis* plants, but were reduced in the *TaSERK5*–OE line. Unlike *LEC2*, *WUS* and *AGL–15* the level of expression of *BBM* was drastically reduced. The above results thus suggest that *TaSERKs*-OE in *Arabidopsis* alters the expression of zygotic genes specifically in the siliques, implying the coordination of somatic and zygotic genes during seed development.

## Discussion

### Structural similarity of *TaSERK* genes

The plant RLKs form a large gene family comprises of more than 600 members in *Arabidopsis* and almost 300 LRR–RLKs in rice^41,42^. Amongst the SERK proteins identified so far, the most extensively studied was the *Arabidopsis AtSERK1* which plays a pleiotropic role^5,43–46^. Sequence and structural analyses indicated that TaSERK1, 2, 3, 4 and 5 encode a typical SERK protein belonging to the LRR–RLK family^47^. The predicted domain structure of TaSERKs consists of a signal peptide, leucine zipper (LZ) region (absent in TaSERK4), five LRR domains, a characteristic SPP motif, a single transmembrane domain, serine/threonine kinase domain and a highly conserved C–terminal domain which are very similar to other characterized SERKs including AtSERK1^5^, OsSERK1 and OsSERK2^30^, and ZmSERK1^21^. The five LRR repeats in the SERK domain structure form a horseshoe-shaped cavity predicted to be involved in protein-protein interactions during molecular recognition processes in animals and plants^48^. TaSERK1, 2, 3, 4 and 5 were recently found to interact with TaBRI1 at the plasma membrane^49^. The hallmark of SERK proteins is the presence of a SPP motif that acts as a hinge providing flexibility to the extracellular part of the receptor^5^. The sequence similarity of TaSERK1, TaSERK2, TaSERK3, TaSERK4 and TaSERK5 with AtSERK1 and AtSERK2 implies that the corresponding TaSERKs may play similar functional roles during morphogenesis, cell signaling and plant development. The C–terminal leucine rich domain highly conserved in SERK proteins plays a key role in protein–protein interactions^4^. Alignment of the five TaSERKs reveals differential amino acid sequences present in the signal peptide, with TaSERK2, TaSERK3 and TaSERK4 lacking some amino acid residues compared to the TaSERK1 and TaSERK5. Some differences in amino acids were also located in the SPP region of TaSERK3 and TaSERK4. Differences in the C–terminal domain may be responsible for differential expression and function. Phylogenetic studies reveal that TaSERK1 and TaSERK5 may perform a similar function as they clustered together. TaSERK2 and TaSERK3 also cluster together while TaSERK4 was found to be more closely associated with ZmSERK1^21^, indicating that TaSERKs may play a redundant role in SE and have various functional aspects as observed for other plant species. The sequence analyses and phylogenetic tree reveal that the conservation of TaSERKs is very extensive within a subgroup encompassing the monocot OsSERK1, OsSERK2, ZmSERK1, ZmSERK2 and ZmSERK3 and the dicot AtSERK1, AtSERK2, PpSERK1, PpSERK2, MtSERK2 and MtSERK5.

### Expression of *TaSERKs* correlates in different tissues and organs

There are various reports on the probable role of *SERKs* in different aspects of plant development. In the present study, we analysed the expression profile of *TaSERKs* revealing their participation both in somatic as well as zygotic embryogenesis. Amongst all five *TaSERK* members, the expression pattern analyses of *TaSERK1* and *TaSERK4* appears to implicate them specifically in SE rather than in zygotic tissues, thus suggesting specificity to SE similar to the *OsSERK1* and *OsSERK2* ^30^. Interestingly, *TaSERK2* and *TaSERK5* are highly expressed in ovary tissues and spikes in contrast to *TaSERK*3 which showed high abundance of transcript in anthers but low abundance in ovaries, respectively implies that these may play a significant role during zygotic embryogenesis, consistent with the results of *AtSERK1*, *AtSERK2, MtSERK1 andCitSERK1* ^22,31,50,51^. The high expression level of *TaSERK1*, *TaSERK2* and *TaSERK4* in embryogenic callus specifically under light culture conditions with low expression in non–embryogenic callus both under light and dark conditions, suggest a functional similarity to *ZmSERK1* and *ZmSERK2* genes which were reported to be expressed both in embryogenic and non–embryogenic callus cultures^21^. The *SERK* expression patterns^28^ were also reported in, callus tissues of *Dactylis glomerata* ^20^ and *Arabidopsis thaliana* ^5^, validating the indispensable role of SERKs during SE in these plants. Thus, the above expression patterns corroborate that the function of the wheat *SERK* gene family members is not only restricted to embryogenesis but they play a crucial role in other organs at major developmental stages.

The expression of the five *TaSERKs* was also monitored under the influence of phytohormones, auxin and BR. The transcript level of *TaSERK1* and *TaSERK2* showed upregulation upon induction with 2,4–D as compared to *TaSERK*3, 4 and *5*. In contrast, *TaSERK1* and *TaSERK5* were induced significantly to a high extent in the presence of BR. These results suggest that the *TaSERK2* response is highly auxin mediated compared to other *TaSERKs*, while *TaSERK1* and *TaSERK5* may be mediated by the BR signaling pathway, consistent with *AtSERK1*, *2*, *3* and *4* ^52^.

### Constitutive expression of *TaSERKs* regulate root and hypocotyl growth in *Arabidopsis*

Earlier reports and results from the present study clearly indicate that SERKs also regulate root development. It was reported that auxins play a key role in root development of *Arabidopsis* plants^53–55^ and together with BRs also participate in regulating the growth largely through mediating control over essential genes^56–59^. Evidence from genetic mutant analysis validates the role of SERKs regulating root development primarily via a BR–independent pathway as the triple mutant *serk2bak1bkk1* exhibited shortened root phenotype that was rescued by the expression of *SERK1*, *BAK1/SERK3* and *BKK1/SERK4*, respectively^60^. OE of each of the five *TaSERKs* enhanced primary root growth in *Arabidopsis* and also elicited sensitivity to the inhibitory action of exogenously supplied auxin and brassinosteroid hormones in a dose-dependent manner, suggesting their functional role in the development and an enhancement of root growth at lower concentrations of BR in an auxin-independent manner. Nonetheless, it is still unclear how the *TaSERKs* modulate root development when induced in combination with auxin and BR.

The OE of *TaSERK 1*, *2*, *3*, *4* and *5* led to the diverse changes in the growth and morphology of *Arabidopsis*. The major alterations were observed in plant height, silique size and seed yield. In contrast, the constitutive expression of *AtSERK1* did not show any altered phenotype; however, seedlings which overexpressed *AtSERK1* initiated SE with higher efficiency^5^. Transgenic plants harbouring *TaSERK1*, *TaSERK2*, *TaSERK3*, *TaSERK4 and TaSERK5* independently displayed common alterations in plant height, rosette and leaf development, silique morphology, seed yield, and seed size. The phenotypic observations suggest that enhanced expression of *TaSERK* family members exhibit overlapping functions in *Arabidopsis* transgenics.

### *TaSERK* enhances the expression of zygotic genes in *Arabidopsis*

A small set of transcription factor (TF) specific genes reported from earlier experimental studies which play an essential role in the embryogenic development includes *LEAFY COTYLEDON*(*LEC*)^8,61^, *WUSCHEL* (*WUS*)^13^, *BABY BOOM* (*BBM*)^6^ and *AGAMOUS–LIKE15* (AGL15)^62^. The *LEC* genes are presumed to link the maturation phase of zygotic embryogenesis (ZE) and initiation of SE via the establishment of a suitable environment for cell differentiation^63^. *LEC1* expression in *Arabidopsis* embryogenesis culture is also involved in differentiation and development apart from somatic embryo induction^64^. *Arabidopsis LEC1* was found to be expressed at higher levels in siliques during early embryo development compared to maturing embryos^65^. In our study, *LEC1* transcript expression correlated with a significant increase in siliques on transgenic *TaSERK2*, *TaSERK3* and *TaSERK1* plants, suggesting a link between *SERK* gene family members and *LEC* in regulating gene expression during the maturation phase of embryo development. The *WUS* gene in *Arabidopsis* promotes the transition from vegetative to embryonic phase in all tissues and organs^13^. Here, we show that the ectopic expression of *TaSERKs* in *Arabidopsis* results in increased expression of *WUS* in siliques and lower expression in flowers, indicating that constitutively expressing *TaSERK1*, *2* and *3* modulate zygotic embryo development. Further, some studies have reported the expression of BBM during zygotic and pollen–derived SE^6^. In our study, we show that *TaSERK1*, *2* and *3* transgenic plants exhibit higher expression of *BBM* specifically in siliques, suggesting ectopic expression of *TaSERK1*, *2* and *3* in *Arabidopsis* may regulates zygotic embryo development. We also examined the expression pattern of AGL–15, a MADS domain TF reported to play a role in embryogenesis. *AGL–15* was found to be expressed during early zygotic embryogenesis^66,67^ and its ectopic expression upregulated *AtSERK1* expression^62^. The higher expression of *AGL–15* in *TaSERK1*, *2*, *3* and *4 Arabidopsis* transgenics suggests a correlation between *SERK* and zygotic genes. *TaSERK5* transgenics, however, do not participate in the regulation of zygotic embryo related gene expression in plant development. Nevertheless, the expression levels of *LEC1*, *WUS*, *BBM*, and *AGL-15* were significantly affected in silique tissues of *TaSERK* transgenic plants, indicating that they may be involved in the regulation of seed development.

## Conclusion

*TaSERK* gene family members are functionally redundant based on phenotypic observations as well as gene expression patterns in transgenic lines. *TaSERKs* display sequence conservation and exhibit differential expression in zygotic and somatic tissues of wheat. The effect of auxin and brassinosteroid on tissue specific expression and in root growth suggests the involvement of *TaSERKs* in hormonal regulation pathway. Taken together, the findings from the present study confirms that *TaSERKs* exert a profound effect on embryogenesis, plant growth and development, thus suggesting wide- range function of *TaSERK* genes which were earlier restricted to their role in SE.

## Electronic supplementary material

Supplementary File
