# Supplementary material for: Ectopic expression of Triticum aestivum SERK genes (TaSERKs) control plant growth and development in Arabidopsis
Source: Sci Rep. 2017 Sep 28;7:12368. doi: 10.1038/s41598-017-10038-1 (PMC5620050; doi:10.1038/s41598-017-10038-1)
Supplement: Supplementary file 1 — Supplementary File [file 41598_2017_10038_MOESM1_ESM.pdf]

**Ectopic expression of *Triticum aestivum SERK* genes (TaSERKs) control plant growth and development in *Arabidopsis***

Akanksha Singh and Paramjit Khurana*

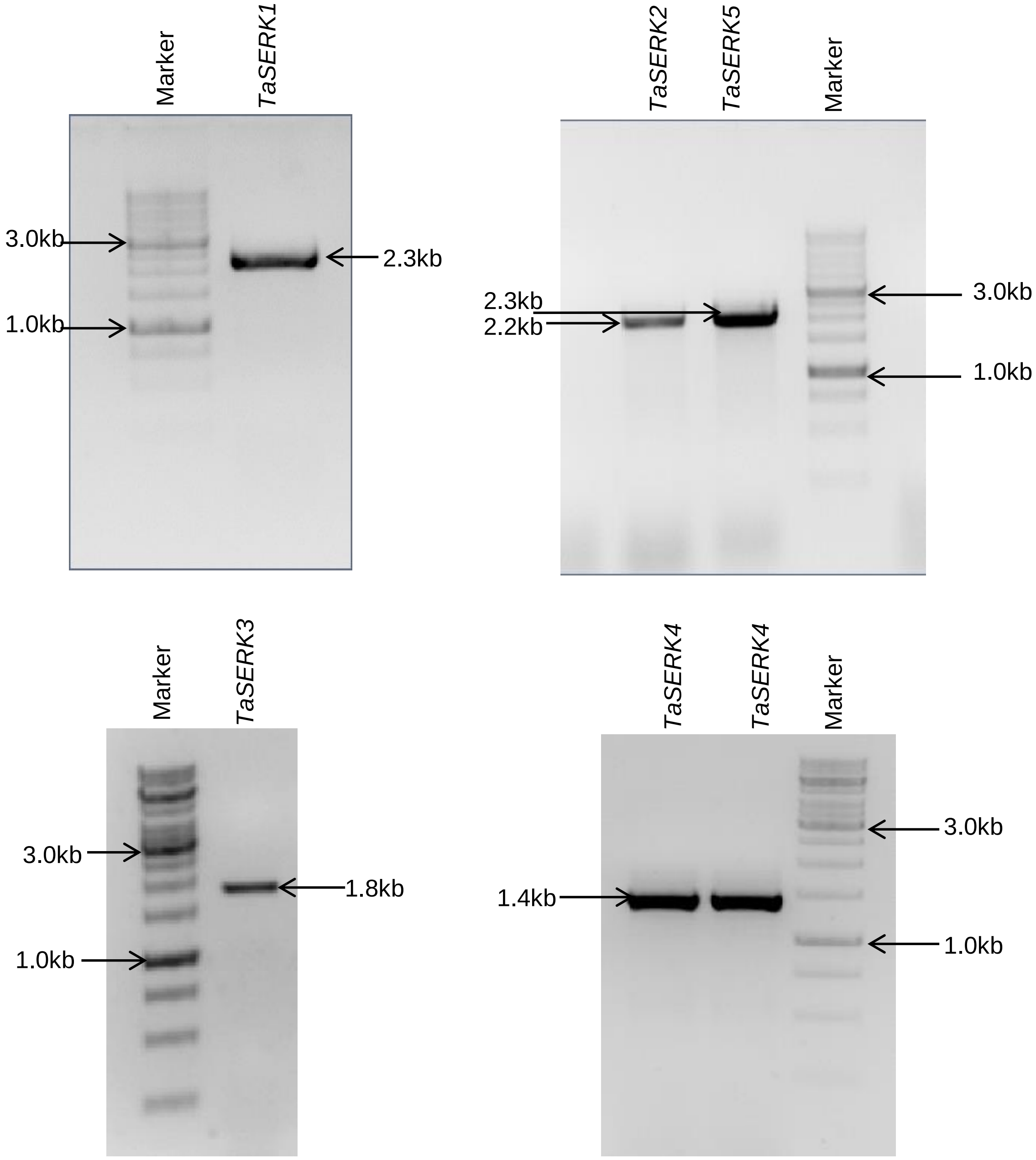


**Supplementary Fig. S1. Amplified PCR product of TaSERKs from *T. aestivum.*** Purified PCR product of *TaSERK1* (2.3kb), *TaSERK2* (2.2 kb), *TaSERK3* (1.8kb), *TaSERK4* (1.4kb) and *TaSERK5* (2.3kb) amplified from cDNA which was isolated from embryogenic calli of wheat (*T. aestivum*).

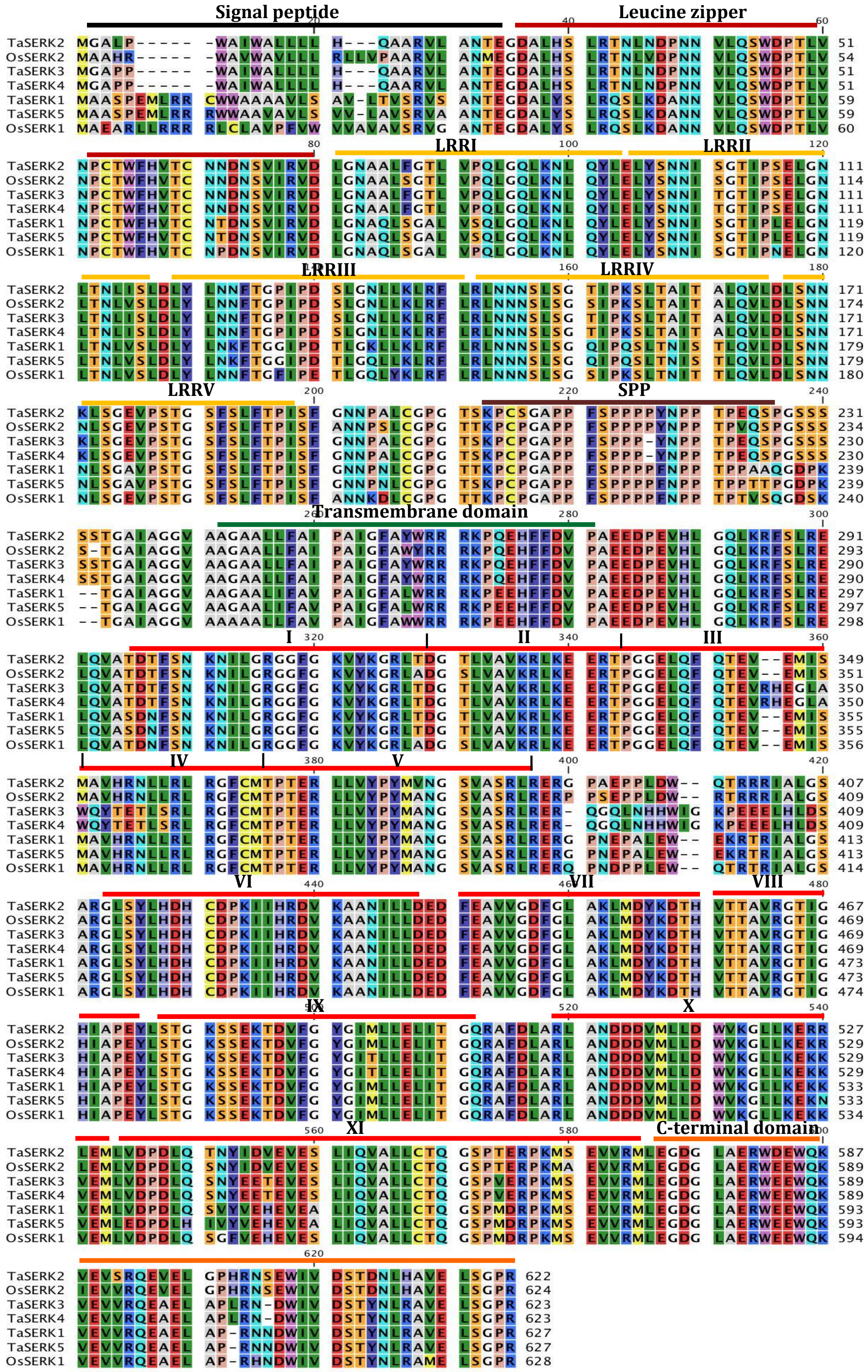


**Supplementary Fig. S2.** Multiple sequence alignment of TaSERK1, 2, 3, 4 and 5 with OsSERK1, 2. The different domains are labelled and shown by coloured lines.

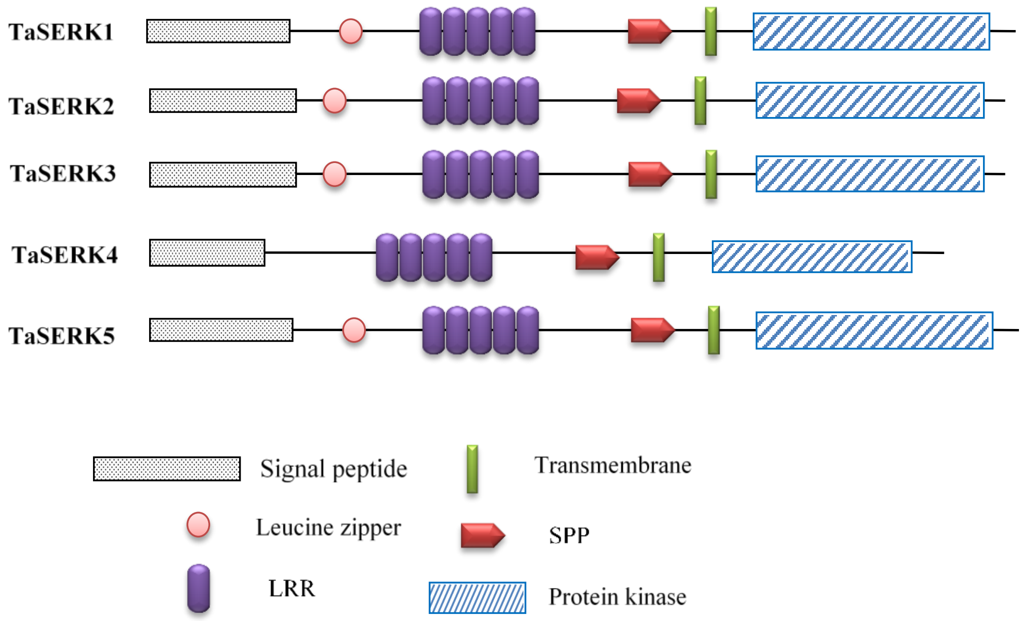


**Supplementary Fig. S3.** Domain analysis of TaSERKs.

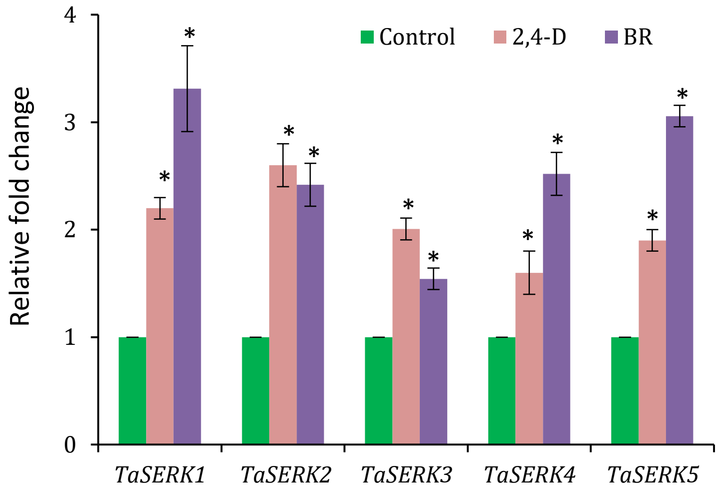


**Supplementary Fig. S4. Expression analysis of five *TaSERKs* in control, 2,4-D and epibrassinolide treated leaf bases of 13-day-old wheat seedlings.** The relative mRNA levels were normalised with respect to the housekeeping gene, *ACTIN*. The error bars represent mean ± SD of two biological replicates, each analyzed with three technical replicates. The asterisks above the bars indicate statistically significant differences at *$p \leq 0.05$.

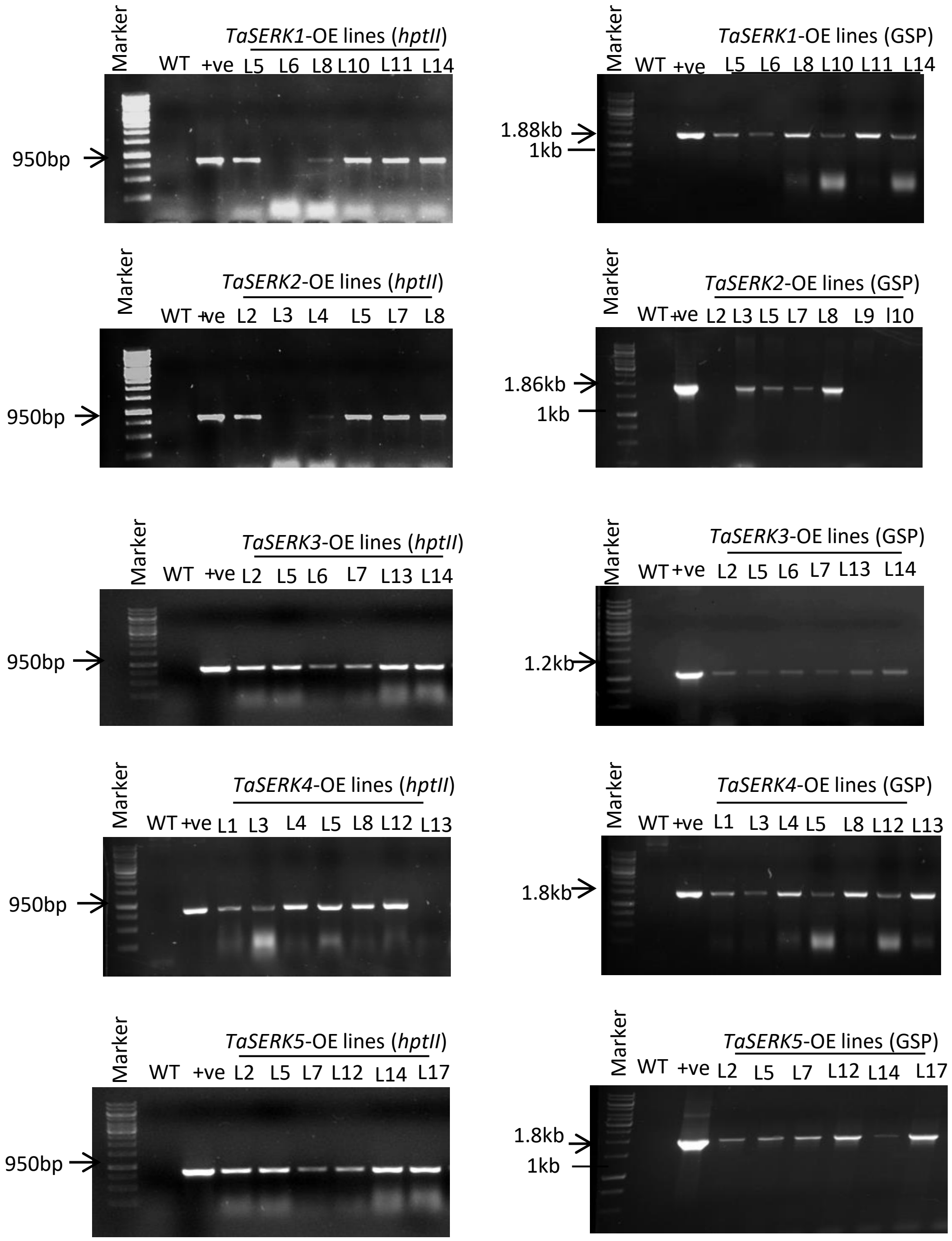


**Supplementary Fig. S5.** Confirmation of *TaSERK1,2,3,4* and *5* transgenics. Confirmation of putative transformants of *TaSERK*-OE transgenics (*TaSERK1,2,3,4 and 5*) in *Arabidopsis* through PCR using *hptII* and *TaSERKs* gene specific primers.

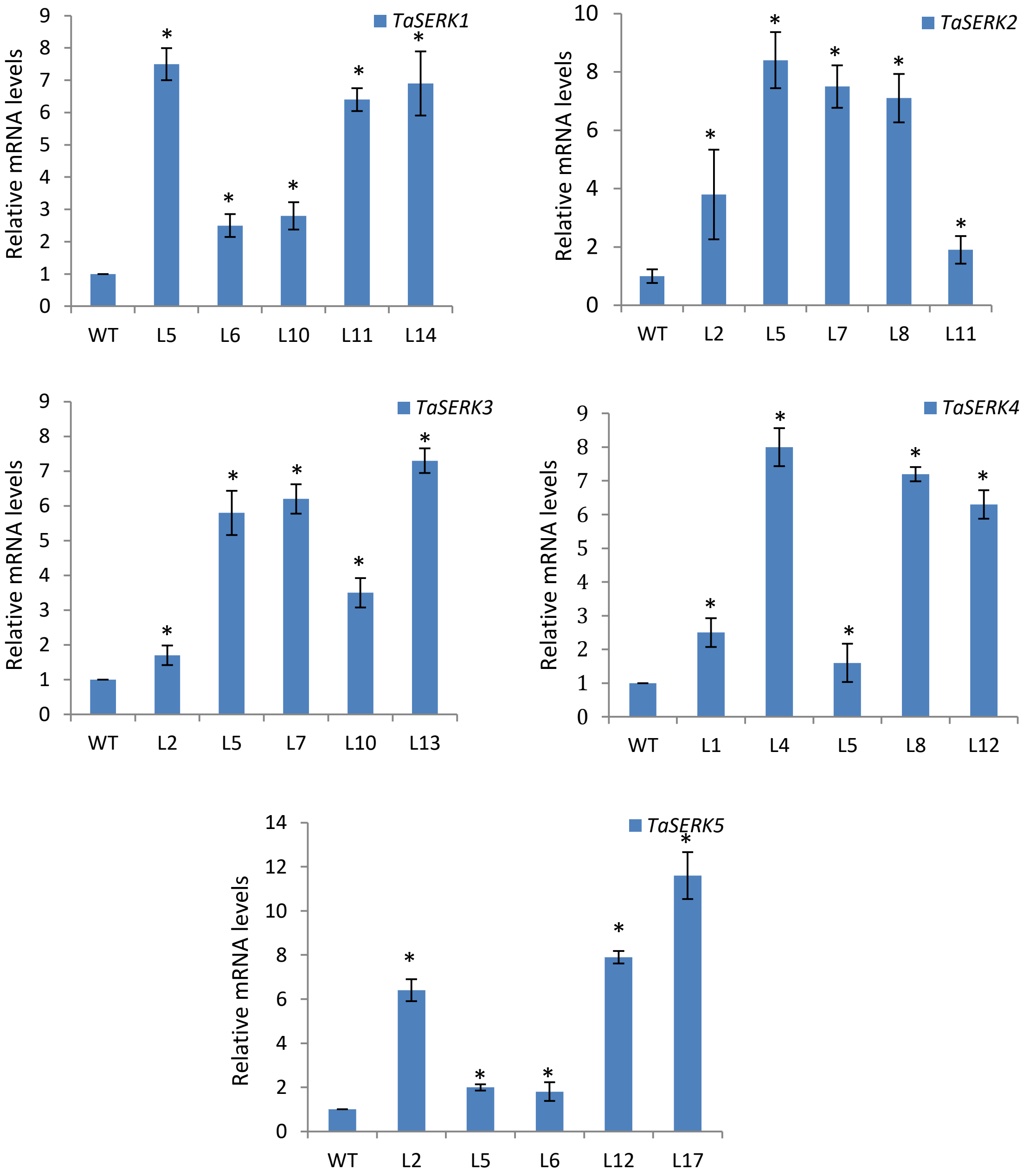


**Supplementary Fig. S6.** Overexpression of all five *TaSERK*s in *Arabidopsis* transgenic plants. Quantitative PCR analysis of WT and transgenic *Arabidopsis* plants, representing the transcript level of *TaSERK1, 2, 3, 4* and *5* relative to the WT. The expression of *ACTIN* gene was used as an internal control. The Data showed are mean ± SE from two biological replicates. The asterisks above the bars indicate statistically significant differences at *p≤ 0.05.

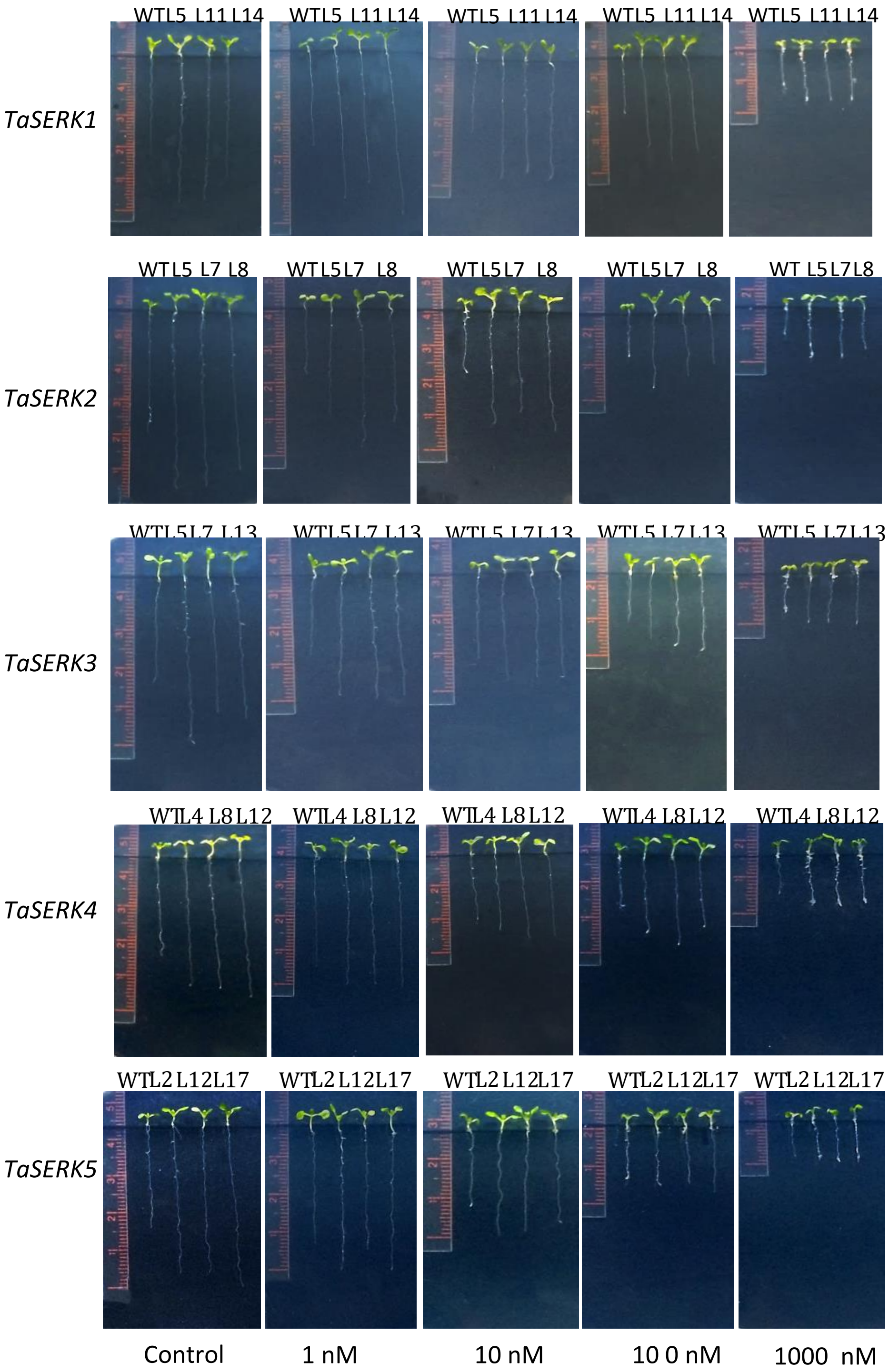


**Supplementary Fig. S7.** Root growth elongation assay of *TaSERK1, 2, 3, 4* and *5* OE plants under 2,4-D treatment. 7-d-old seedlings of WT and *TaSERKs*-OE were grown on half-strength MS medium in the presence or absence of the indicated concentration of 2,4-D (nM) after germination.

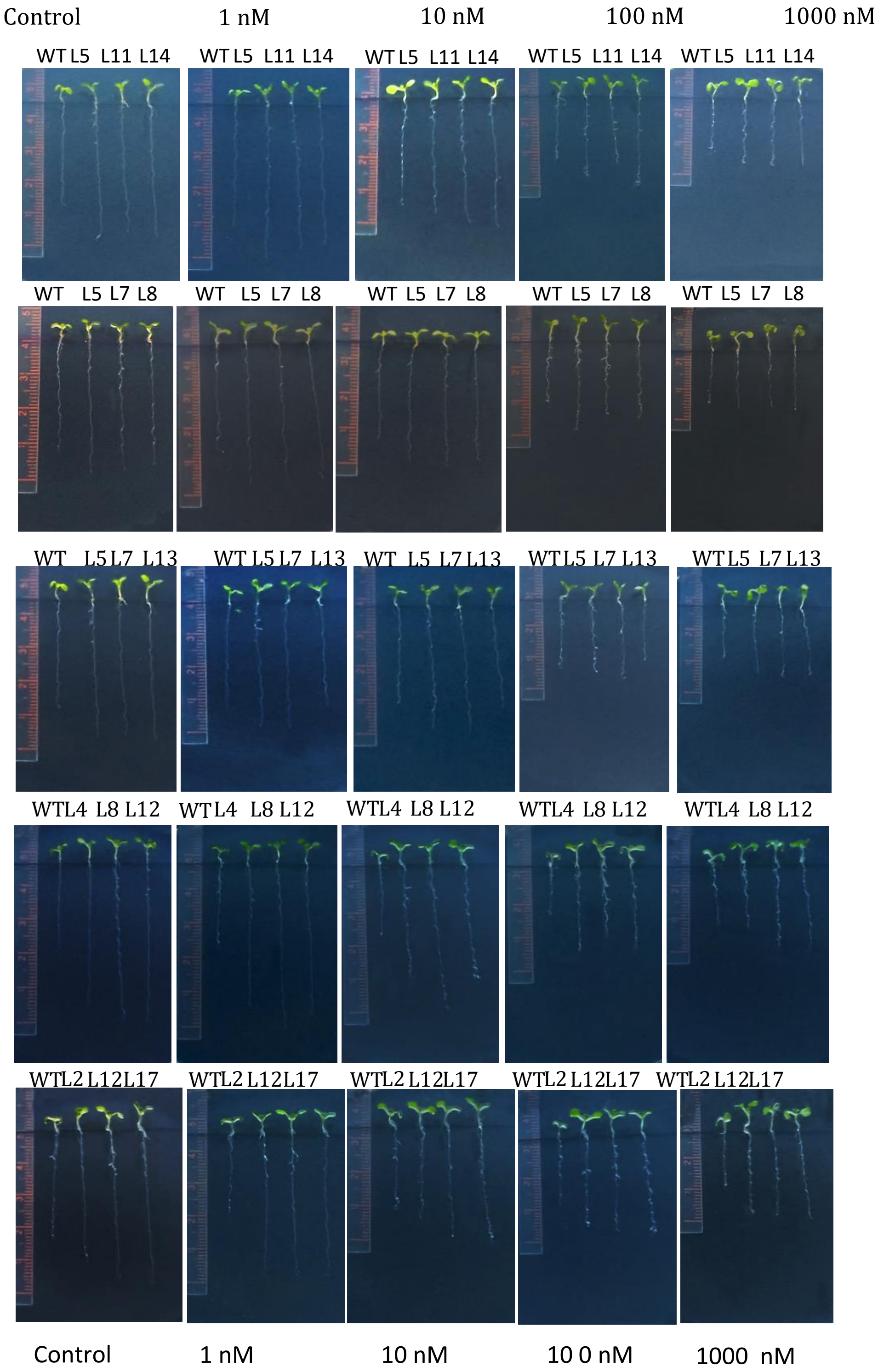


**Supplementary Fig. S8.** Root growth elongation assay of *TaSERK1*, *2*, *3*, *4* and *5* OE plants under epi-BL treatment. 7-d-old seedlings of WT and *TaSERKs*-OE were grown on half-strength MS medium in the presence or absence of the indicated concentration of epi-BL (nM) after germination.

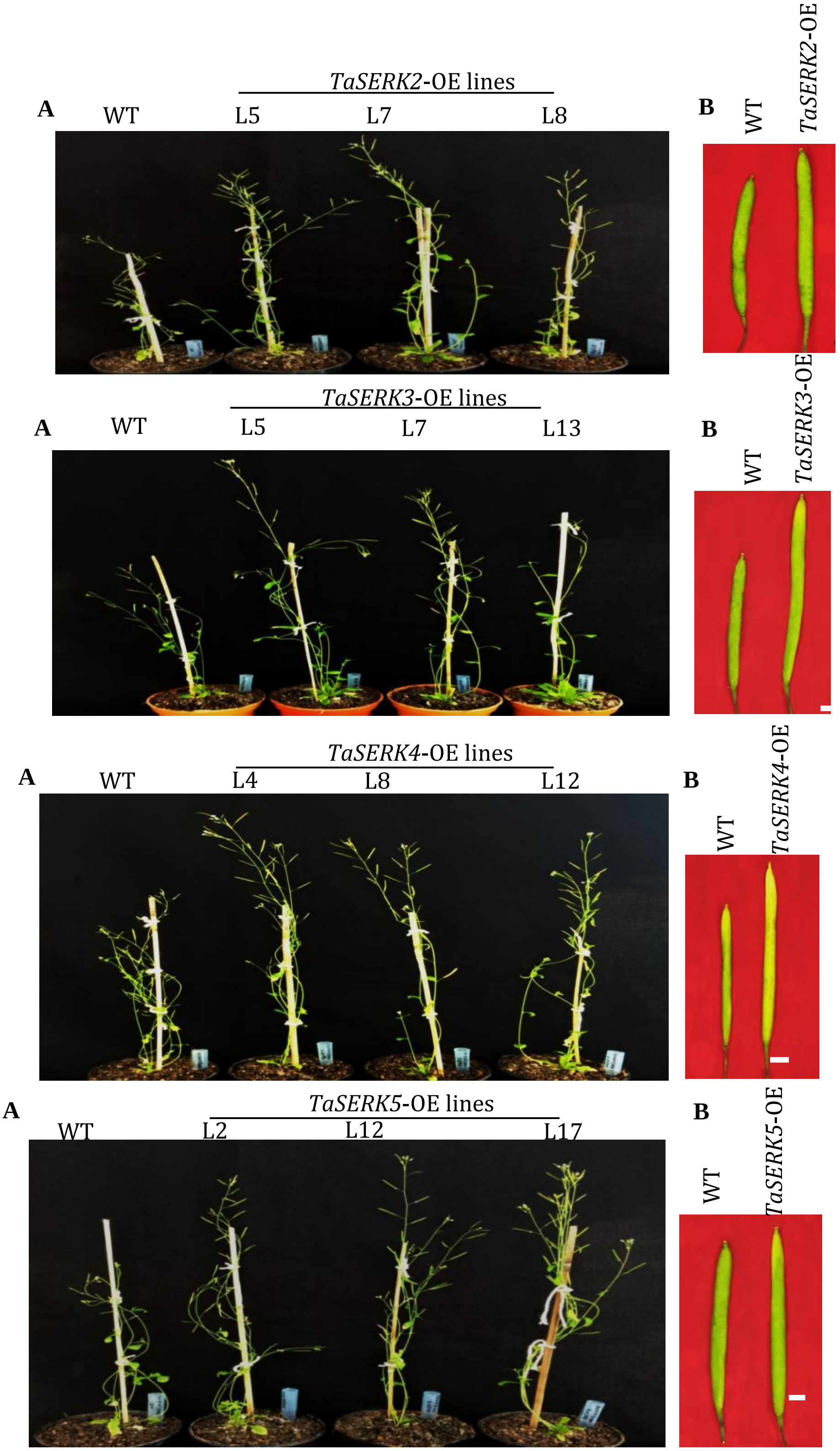


**Supplementary Fig. S9.** Phenotype of *TaSERKs* OE transgenic lines in *Arabidopsis thaliana.* **A.** *TaSERK2, 3, 4* and 5 OE transgenic lines showed increase in plant height as compared to the Col-0 WT grown under 16 h light and 8 h dark culture condition at the same time. **B.** Two month old *TaSERK*s-OE lines had slight increased siliques than WT.

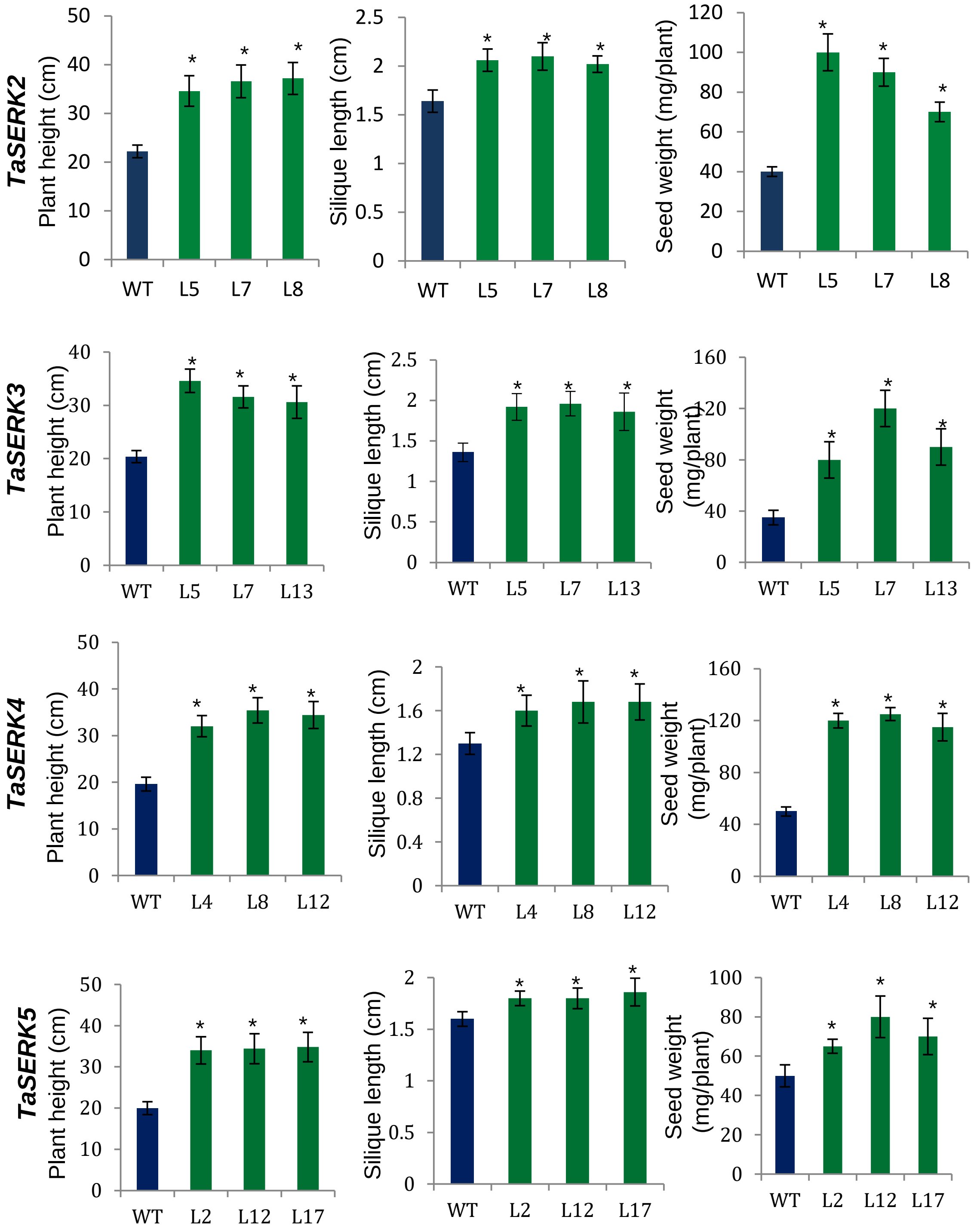


**Supplementary Fig. S10. Morphometric comparison of *TaSERK2, 3, 4* and *5* overexpression transgenic lines in *Arabidopsis thaliana*.** Graphical representation of plant height, silique length and seed weight per individual plant (N=10). Data represents mean ±SE. The asterisks (**p*≤0.05) indicate statistically significant differences between WT and transgenic lines.

**Supplementary Table S1.** Morphological comparison between *TaSERK1*, 2, 3, 4 and *5* OE and WT *Arabidopsis* plants.

| Morphological features of *TaSERK1* | WT | L5 | L11 | L14 |
|---|---|---|---|---|
| Leaf length[a] (cm) | 1.65 ± 0.18 | 1.72 ± 0.11 | 1.7 ± 0.11 | 1.74 ± 0.11 |
| Leaf width[a] (cm) | 0.56 ± 0.01 | 0.57 ± 0.02 | 0.58 ± 0.01 | 0.57 ± 0.03 |
| Rosette leaf no.[b] | 10.8 ± 0.83 | 6.8 ± 0.84 | 7.2 ± 0.84 | 7.8 ± 1.30 |
| Silique number | 16 ± 3.08 | 36.4 ± 4.66 | 31.8 ± 5.31 | 33.8 ± 3.70 |
| Seed length | 0.35 ± 0.02 | 0.5 ± 0.01 | 0.44 ± 0.03 | 0.50 ± 0.03 |
| Seed width | 0.24 ± 0.03 | 0.29 ± 0.02 | 0.35 ± 0.04 | 0.31 ± 0.05 |

| Morphological features of *TaSERK2* | WT | L5 | L7 | L8 |
|---|---|---|---|---|
| Leaf length[a] (cm) | 1.6 ± 0.07 | 1.69 ± 0.05 | 1.7 ± 0.10 | 1.7 ± 0.08 |
| Leaf width[a] (cm) | 0.48 ± 0.01 | 0.68 ± 0.05 | 0.64 ± 0.01 | 0.54 ± 0.04 |
| Rosette leaf no.[b] | 10.2 ± 0.83 | 8.4 ± 1.14 | 7.4 ± 0.83 | 7.4 ± 0.54 |
| Silique number | 19.6 ± 2.30 | 28.8 ± 2.86 | 36.6 ± 5.07 | 44.6 ± 4.61 |
| Seed length | 0.30 ± 0.02 | 0.49 ± 0.03 | 0.54 ± 0.02 | 0.51 ± 0.04 |
| Seed width | 0.23 ± 0.02 | 0.31 ± 0.05 | 0.32 ± 0.04 | 0.35 ± 0.04 |

| Morphological features of *TaSERK3* | WT | L5 | L7 | L13 |
|---|---|---|---|---|
| Leaf length[a] (cm) | 1.52 ± 0.08 | 1.74 ± 0.11 | 1.78 ± 0.13 | 1.72 ± 0.11 |
| Leaf width[a] (cm) | 0.52 ± 0.04 | 0.74 ± 0.05 | 0.7 ± 0.07 | 0.72 ± 0.08 |
| Rosette leaf no.[b] | 11.6 ± 1.51 | 9 ± 0.70 | 9.2 ± 0.83 | 10 ± 1.58 |
| Silique number | 20.6 ± 1.92 | 34.6 ± 4.15 | 35.6 ± 3.84 | 31.8 ± 5.31 |
| Seed length | 0.41 ± 0.01 | 0.55 ± 0.02 | 0.50 ± 0.03 | 0.47 ± 0.05 |
| Seed width | 0.28 ± 0.03 | 0.36 ± 0.04 | 0.31 ± 0.04 | 0.32 ± 0.03 |

| Morphological features of *TaSERK4* | WT | L4 | L8 | L12 |
|---|---|---|---|---|
| Leaf length[a] (cm) | 1.38 ± 0.08 | 1.6 ± 0.07 | 1.56 ± 0.11 | 1.62 ± 0.08 |
| Leaf width[a] (cm) | 0.48 ± 0.02 | 0.58 ± 0.01 | 0.56 ± 0.02 | 0.6 ± 0.03 |
| Rosette leaf no.[b] | 11.4 ± 1.67 | 8.8 ± 1.48 | 8.8 ± 0.83 | 8.6 ± 1.14 |
| Silique number | 23.6 ± 3.36 | 35.0 ± 4.30 | 44.2 ± 5.17 | 38.8 ± 2.58 |
| Seed length | 0.38 ± 0.03 | 0.47 ± 0.02 | 0.52 ± 0.06 | 0.45 ± 0.04 |
| Seed width | 0.26 ± 0.04 | 0.34 ± 0.03 | 0.31 ± 0.05 | 0.30 ± 0.02 |

| Morphological features of *TaSERK5* | WT | L2 | L12 | L17 |
|---|---|---|---|---|
| Leaf length[a] (cm) | 1.5 ± 0.12 | 1.64 ± 0.18 | 1.58 ± 0.13 | 1.68 ± 0.13 |
| Leaf width[a] (cm) | 0.56 ± 0.01 | 0.62 ± 0.02 | 0.64 ± 0.01 | 0.6 ± 0.01 |
| Rosette leaf no.[b] | 10.6 ± 1.14 | 8.6 ± 1.14 | 8.6 ± 1.14 | 8.4 ± 1.51 |
| Silique number | 20.2 ± 1.30 | 32.0 ± 2.54 | 40.6 ± 3.04 | 41.2 ± 4.76 |
| Seed length | 0.40 ± 0.04 | 0.45 ± 0.04 | 0.50 ± 0.03 | 0.47 ± 0.06 |
| Seed width | 0.31 ± 0.02 | 0.33 ± 0.02 | 0.31 ± 0.04 | 0.32 ± 0.05 |

**Supplementary Table S2.** List of primers used in the experiment

| Gene | Primer ID | Sequence (5'- 3') |
|---|---|---|
| *TaSERK1* | TOPO-Fwd | CACCATGGCTGCGTCGCCGGAGATG |
| | TOPO-Rev | TCCTGTTACCTCGGGCCGGACAGCTC |
| | Fwd (Real Time) | CACCGCCTCCATTCAATCC |
| | Rev (Real Time) | CTCCGGTTTTAGGGTCACCTT |
| *TaSERK2* | TOPO-Fwd | CACCATGGGGGCGCTGCCGTGG |
| | TOPO-Rev | CAGCCACCATCACCTTGGCCCTGATAG |
| | Fwd (Real Time) | AAGTGTTGCATCACGTCTAAGAG |
| | Rev (Real Time) | GGCAGAACCCAATGCAATTC |
| *TaSERK3* | TOPO-Fwd | CACCTGCTGTGGGATCTAGGGC |
| | TOPO-Rev | AGGCTGAATGACTGATCAAATGGTG |
| | Fwd (Real Time) | TTCGGCACGAGGGATTAGC |
| | Rev (Real Time) | CATTGGCCATGTATGGATAGACTAGT |
| *TaSERK4* | TOPO-Fwd | CACCATGTCGGGTCAAATTCCTAA |
| | TOPO-Rev | CCTATGCCAGGACGTTGGTCAT |
| | Fwd (Real Time) | GACTCTCTTACTTGCACGATCACTGT |
| | Rev (Real Time) | TCCTCGTCCAAAAGAATATTTGC |
| *TaSERK5* | TOPO-Fwd | CACCATGGCTGCGTCGCCGGAGAT |
| | TOPO-Rev | TCCTGTTACCTCGGGCCGGACAGCT |
| | Fwd (Real Time) | ATCACTGGACAGAGGGCATTTG |
| | Rev (Real Time) | CCCAGTCAAGCAGCATGACA |
| *AtLEC2* | Fwd (Real Time) | GAGCAAGCTTGGGTTCGATAA |
| | Rev (Real Time) | GTCTCTATCTCACGGTACCGGTTAA |
| *AtWUS* | Fwd (Real Time) | GGCTCGTGAGCGTCAGAAG |
| | Rev (Real Time) | AACCGAGTTGGGTGATGAAGA |
| *AtBBM* | Fwd (Real Time) | AGTGACGAGTTCGCGTTTGG |
| | Rev (Real Time) | AGACATGTGTATGTGTGAACGTATGAC |
| *AtAGL-15* | Fwd (Real Time) | CCACTATGTTCCATCCTACATCAAA |
| | Rev (Real Time) | GGTGTTCTGGAGGCTGCATT |
